# Subtype cluster analysis unveiled the correlation between m6A- and cuproptosis-related lncRNAs and the prognosis, immune microenvironment, and treatment sensitivity of esophageal cancer

**DOI:** 10.3389/fimmu.2025.1539630

**Published:** 2025-02-17

**Authors:** Ming Zhang, Yani Su, Pengfei Wen, Xiaolong Shao, Peng Yang, Peng An, Wensen Jing, Lin Liu, Zhi Yang, Mingyi Yang

**Affiliations:** ^1^ Department of General Practice, Honghui Hospital, Xi’an Jiaotong University, Xi’an, China; ^2^ Department of Joint Surgery, Honghui Hospital, Xi’an Jiaotong University, Xi’an, China

**Keywords:** N6-methyladenosine, cuproptosis, lncRNA, prognosis, immune, esophageal cancer

## Abstract

**Objective:**

Esophageal cancer (EC) is characterized by a high degree of malignancy and poor prognosis. N6-methyladenosine (m6A), a prominent post-transcriptional modification of mRNA in mammalian cells, plays a pivotal role in regulating various cellular and biological processes. Similarly, cuproptosis has garnered attention for its potential implications in cancer biology. This study seeks to elucidate the impact of m6A- and cuproptosis-related long non-coding RNAs (m6aCRLncs) on the prognosis of patients with EC.

**Methods:**

The EC transcriptional data and corresponding clinical information were retrieved from The Cancer Genome Atlas (TCGA) database, comprising 11 normal samples and 159 EC samples. Data on 23 m6A regulators and 25 cuproptosis-related genes were sourced from the latest literature. The m6aCRLncs linked to EC were identified through co-expression analysis. Differentially expressed m6aCRLncs associated with EC prognosis were screened using the limma package in R and univariate Cox regression analysis. Subtype clustering was performed to classify EC patients, enabling the investigation of differences in clinical outcomes and immune microenvironment across patient clusters. A risk prognostic model was constructed using least absolute shrinkage and selection operator (LASSO) regression. Its robustness was evaluated through survival analysis, risk stratification curves, and receiver operating characteristic (ROC) curves. Additionally, the model’s applicability across various clinical features and molecular subtypes of EC patients was assessed. To further explore the model’s utility in predicting the immune microenvironment, single-sample gene set enrichment analysis (ssGSEA), immune cell infiltration analysis, and immune checkpoint differential expression analysis were conducted. Drug sensitivity analysis was performed to identify potential therapeutic agents for EC. Finally, the mRNA expression levels of m6aCRLncs in EC cell lines were validated using reverse transcription quantitative polymerase chain reaction (RT-qPCR).

**Results:**

We developed a prognostic risk model based on five m6aCRLncs, namely ELF3-AS1, HNF1A-AS1, LINC00942, LINC01389, and MIR181A2HG, to predict survival outcomes and characterize the immune microenvironment in EC patients. Analysis of molecular subtypes and clinical features revealed significant differences in cluster distribution, disease stage, and N stage between high- and low-risk groups. Immune profiling further identified distinct immune cell populations and functional pathways associated with risk scores, including positive correlations with naive B cells, resting CD4+ T cells, and plasma cells, and negative correlations with macrophages M0 and M1. Additionally, we identified key immune checkpoint-related genes with significant differential expression between risk groups, including TNFRSF14, TNFSF15, TNFRSF18, LGALS9, CD44, HHLA2, and CD40. Furthermore, nine candidate drugs with potential therapeutic efficacy in EC were identified: Bleomycin, Cisplatin, Cyclopamine, PLX4720, Erlotinib, Gefitinib, RO.3306, XMD8.85, and WH.4.023. Finally, RT-qPCR validation of the mRNA expression levels of m6aCRLncs in EC cell lines demonstrated that ELF3-AS1 expression was significantly upregulated in the EC cell lines KYSE-30 and KYSE-180 compared to normal esophageal epithelial cells.

**Conclusion:**

This study elucidates the role of m6aCRLncs in shaping the prognostic outcomes and immune microenvironment of EC. Furthermore, it identifies potential therapeutic agents with efficacy against EC. These findings hold significant promise for enhancing the survival of EC patients and provide valuable insights to inform clinical decision-making in the management of this disease.

## Introduction

Esophageal cancer (EC) is a highly aggressive malignancy with a poor prognosis, representing a significant global health challenge (1). Despite advances in treatment modalities—including surgery, chemotherapy, radiotherapy, molecular targeted therapy, and various combination approaches—both the morbidity and mortality rates associated with EC remain alarmingly high (2, 3). These therapeutic advancements have yet to achieve substantial improvements in long-term outcomes, as the overall prognosis for EC patients remains grim (4). Consequently, EC continues to pose a serious threat to public health worldwide, underscoring the urgency of developing more effective therapeutic strategies (5, 6). Addressing these challenges requires an integrated effort to better understand the disease’s molecular mechanisms and to identify innovative treatments capable of improving patient survival and quality of life.

Copper is an essential cofactor required for the survival and function of all organisms, playing critical roles in various biochemical processes. However, when copper concentrations exceed the levels maintained by evolved homeostatic systems, it becomes toxic (7). This toxicity is primarily mediated through the disruption of the tricarboxylic acid (TCA) cycle, where copper directly binds to the lipid components of TCA cycle enzymes, inducing copper-dependent cell death (cuproptosis). In cells with active TCA cycles, elevated levels of lipid-acylated TCA enzymes and direct copper binding to their lipid acyl moieties result in the accumulation of lipid-acylated proteins, depletion of Fe-S cluster-containing proteins, and activation of HSP70. These events collectively lead to protein stress and ultimately cellular demise (7). Recent studies have identified cuproptosis-related genes (CRGs) as key determinants in predicting prognosis for certain cancers, including colorectal cancer and osteosarcoma, using risk prognostic models (8–12). These findings underscore the potential of CRGs as novel biomarkers and therapeutic targets. However, the role of CRGs in EC has not been further explored, representing a critical gap in the understanding of copper-induced cellular toxicity and its implications for EC prognosis and treatment. Further investigation is warranted to elucidate the function of CRGs in EC and their potential utility in clinical practice.

N6-methyladenosine (m6A) represents the most prevalent internal modification of mRNA in mammalian cells, playing a pivotal role in post-transcriptional regulation (13). First identified in the 1970s (14), m6A has since been recognized as a crucial regulator of diverse cellular and biological processes (14). This modification influences the structural conformation, stability, degradation, and cellular interactions of mRNA, thereby modulating key processes such as splicing, translation, nuclear export, and RNA decay (15). The implications of m6A methylation extend far beyond fundamental biology, with growing evidence highlighting its critical role in human diseases. Aberrant m6A modification has emerged as a hallmark of cancer, with methylation patterns of m6A-related genes offering potential as distinctive diagnostic biomarkers and therapeutic targets (16). Moreover, m6A plays a vital role in regulating tumor metabolism, further underscoring its significance in cancer progression and treatment strategies (17). As research continues to unravel the complexities of m6A modifications, their potential to transform cancer diagnostics and therapeutics becomes increasingly apparent. Exploring the intricate interplay between m6A methylation and disease processes will likely yield novel insights and open new avenues for precision medicine.

The regulatory mechanisms involving m6A and cuproptosis have garnered increasing attention in cancer biology due to their multifaceted roles. m6A modifications have demonstrated significant potential not only as prognostic biomarkers but also as key regulators of tumor cell proliferation and immune modulation within the tumor microenvironment (18–20). Similarly, cuproptosis-associated characteristics have been identified as predictive indicators for the prognosis and immune response across various cancer types (21, 22). Importantly, both m6A and cuproptosis are intricately linked to cancer prognosis and are actively involved in shaping the tumor immune microenvironment. Their contributions to immune regulation underscore their potential as therapeutic targets, opening avenues for developing novel immunotherapeutic strategies in oncology. Previous finding underscore the potential of RiskScore system comprising ten m6A/m5C-related lncRNAs as effective biomarkers for predicting survival outcomes, characterizing the immune landscape, and assessing response to immunotherapy in esophageal squamous cell carcinoma (ESCC) (23). Study identified that m6A-mediated modification of the autophagy-related gene ATG10 plays a critical role in inducing cuproptosis in kidney chromophobe, revealing a novel intersection between autophagy, m6A methylation, and cuproptosis in cancer biology (24). Previous investigations have extensively examined the prognostic implications of m6A- and cuproptosis-related long non-coding RNAs (m6aCRLncs) across a broad spectrum of malignancies, including hepatocellular carcinoma, head and neck squamous cell carcinoma, gastrointestinal cancers, and clear cell renal cell carcinoma (25–28). These studies collectively underscore the significant value of m6aCRLncs as robust predictors of cancer prognosis, further highlighting their potential to inform clinical decision-making and therapeutic strategies by integrating molecular markers with patient outcomes.

Despite these advancements, the development of a risk prognostic model specifically focused on m6aCRLncs in EC remains unexplored. Given the significant prognostic and therapeutic implications of such models in other malignancies, investigating the role of m6aCRLncs in EC could provide valuable insights. This gap in research underscores the need for future studies to assess the interplay between m6A methylation, cuproptosis, and lncRNAs in EC, potentially unveiling novel biomarkers and therapeutic targets for this aggressive disease. This study successfully established a novel risk prognostic model centered on m6aCRLncs, providing a comprehensive framework for evaluating their critical role in EC. The model highlights the prognostic significance of m6aCRLncs and their potential involvement in modulating the immune microenvironment. By elucidating the interplay between m6aCRLncs and key immunological processes, this research offers valuable insights into their utility not only as predictive biomarkers but also as potential therapeutic targets in EC. Such findings underscore the importance of m6aCRLncs in advancing precision oncology and optimizing treatment strategies for patients with EC.

## Materials and methods

### Data download

The Cancer Genome Atlas (TCGA) database (https://portal.gdc.cancer.gov/) served as the primary source for RNA-sequencing (RNA-seq) data and clinical information related to EC. The dataset included 11 normal samples and 159 EC samples, encompassing both mRNA and lncRNA expression profiles, along with comprehensive clinical annotations for the 159 EC cases. Clinical data were systematically categorized based on survival time (futime), survival status (fustat), gender, clinical stage, and the TNM classification (T, N, and M stages). A set of 25 CRGs was curated from the latest literature (29) and intersected with the gene expression data from the EC microarray to identify EC- related CRGs. Additionally, 23 m6A regulatory genes were extracted from recent publications (30). This forming a foundational dataset for subsequent analyses.

### EC-related CRLncs and m6aCRLncs

The limma package in R was employed to perform co-expression analysis between EC-related CRGs and lncRNAs using the EC RNA-seq dataset, thereby identifying EC-associated CRLncs. A similar approach was utilized to analyze the co-expression of EC-related CRLncs with m6A regulators, resulting in the identification of EC-related m6aCRLncs (31, 32). The selection criteria applied for these analyses were a |Pearson correlation coefficient| > 0.3 and P< 0.001, ensuring a robust statistical threshold for determining significant associations.

### EC prognosis-related differentially m6aCRLncs

Prognosis-related m6aCRLncs in EC were identified through univariate Cox regression analysis, with hazard ratio (HR) values calculated to assess their prognostic significance. Additionally, differential expression analysis of prognosis-related m6aCRLncs in EC was conducted using the limma package in R, comparing 11 normal samples with 159 EC samples. The selection criteria for differentially expressed m6aCRLncs were P< 0.05 and |logFC| > 1. To enhance interpretability, the pheatmap package in R was employed to generate heatmaps visualizing the differential expression profiles, while the ggpubr package in R was used to construct boxplots for detailed visualization of expression differences.

### Analysis of tumor immune microenvironment based on subtype cluster

The “ConsensusClusterPlus” software tool was utilized to classify EC patients into distinct molecular subtypes based on the expression profiles of prognosis-related m6aCRLncs. To evaluate the prognostic implications of these subtypes, survival analysis was performed using the survival and survminer packages in R, assessing differences in overall survival among patients with different subtypes. Clinical feature variations across EC subtypes were analyzed and visualized using the pheatmap package in R. To further investigate the immune microenvironment, the expression data of 22 immune cell types in EC samples were assessed using the CIBERSORT algorithm, identifying immune cell composition differences across subtypes (33–35). Additionally, tumor purity and the stromal and immune cell contributions to the tumor microenvironment were estimated using the ESTIMATE algorithm, which calculates immuneScore, stromalScore, and ESTIMATEScore based on gene expression data (36). These scores were analyzed for subtype-specific differences using the limma package in R.

### Construction of risk prognostic model

LASSO regression analysis was performed using the glmnet package in R to minimize the risk of overfitting and to determine the optimal number of prognosis-related m6aCRLncs for inclusion in the prognostic model. To assess the robustness and predictive accuracy of the model, the dataset was stratified into training (N = 80), testing (N = 79), and overall (N = 159) cohorts. A risk prognostic model was subsequently constructed for each group. The calculation of the riskscore was based on the following formula:


$$Riskscore=\sum_{i=1}^{n}\left(lncrnaexp_{i}\times coef_{i}\right)$$

in the riskscore calculation, n represents the total number of EC prognosis-related m6aCRLncs, while i denotes the individual m6aCRLncs, and coef refers to the corresponding regression coefficient. The riskscore for each sample is determined by multiplying the expression level of each m6aCRLncs by its respective regression coefficient and summing these values (12). Based on the median risk score, the samples from the overall cohort, as well as the training and testing cohorts, were stratified into high-risk and low-risk groups for further analysis.

### Validation of risk prognostic model

Survival analysis was conducted using the survival and survminer packages in R to assess whether there were significant differences in the survival outcomes of EC patients between high-risk and low-risk groups. R was used to generate survival status plot, thereby highlighting the survival rate of patients between the two risk groups. Additionally, the survival and timeROC packages in R were employed to construct Receiver Operating Characteristic (ROC) curves, providing a quantitative measure of the model’s diagnostic accuracy and its potential for risk prediction in EC patients. The pheatmap package in R was used to generate heatmaps depicting of risk scores, thereby highlighting the variations in m6aCRLncs between the two risk groups.

### Difference analysis of clinical features, immunescores and cluster with risk model

The limma package in R was employed to examine whether EC patients with distinct clinical features and subtypes exhibited differences in risk stratification between the high- and low-risk groups within the overall sample cohort. To visualize these differences, the pheatmap package in R was utilized to generate heatmaps, while the ggpubr package was used to create boxplots, allowing for a clear representation of the variations in clinical characteristics and subtypes across the risk groups.

### Immune correlation analysis of risk prognostic model

Single sample gene set enrichment analysis (ssGSEA) was conducted on the risk prognostic model for the overall sample cohort to assess the enrichment scores for immune cells and immune functions in EC patients. To compute these enrichment scores, the limma, GSVA, and GSEABase packages in R were utilized. Differences in immune cell populations and immune function between the high- and low-risk groups were examined using the limma, ggpubr, and reshape2 packages. Additionally, the correlation between the 22 immune cell types and the risk score for the overall sample group was analyzed using the limma, ggpubr, and ggExtra packages in R. Furthermore, to investigate the immune checkpoints that differed between the high- and low-risk groups, the limma, reshape2, and ggpubr packages were applied to the data from the overall sample cohort.

### Drug sensitivity analysis

Drug sensitivity analysis was performed using the limma, pRRophetic, and ggpubr packages in R to identify drugs with varying sensitivities between the high-risk and low-risk groups within the overall sample cohort. This analysis aimed to uncover potential therapeutic agents that could be leveraged to enhance clinical treatment strategies for EC, offering insights into drugs that may exhibit differential efficacy based on risk stratification.

### Cell culture

Human EC cell lines (KYSE-30 and KYSE-180) and normal esophageal epithelial cells (NE-2) were utilized for this study. The EC cell lines (KYSE-30 and KYSE-180) were cultured in RPMI 1640 medium supplemented with 10% fetal bovine serum (FBS), while the normal esophageal epithelial cells (NE-2) were maintained in a Defined Keratinocyte-SFM (DK-SFM) and Epilife mixed medium. All cell cultures were incubated at 37°C in a humidified atmosphere with 5% CO_2_ to promote optimal growth conditions.

### Real-time quantitative PCR

Total RNA was isolated from EC cell lines and normal esophageal epithelial cells (NE-2) using TRIzol Reagent (Cat. No. 15596018, Life Technologies Invitrogen), following the manufacturer’s protocol. The extracted RNA was then subjected to reverse transcription polymerase chain reaction (RT-PCR) using ChamQ Universal SYBR qPCR Master Mix (Cat#: Q711-02, Vazyme), in accordance with the manufacturer’s guidelines, to quantify the mRNA levels of m6aCRLncs. Primer sequences were synthesized by Accurate Biology, and the primer pairs are detailed in **Table 1**. All data were normalized to the expression of β-actin, and relative expression levels were calculated using the 2*^-ΔΔCt^* method.

**Table 1:** Primer sequences for RT-qPCR.

Genes	Forward	Reverse
β-actin	TGGCACCCAGCACAATGAA	CTAAGTCATAGTCCGCCTAGAAGCA
ELF3-AS1	AAAGTTCTTCCCTCAGCGCC	AGTCTGTGCGGTTCGTGATG
HNF1A-AS1	ACTCCAACCCTCTGCTCGTT	AAGTTGCCCAAGGCCATACG
LINC00942	AGCAAGAGAGCGAAGTCCCA	TGTCTTGTGGGAGGCTGACA
LINC01389	CCAAGACTTGATCCCTTGCCC	TATCACTCAGGCCCACACCT
MIR181A2HG	ACCCCCATCCCCTTTTGACA	TCCACAGGACAGTTCGCCTT

## Results

**Figure 1:**
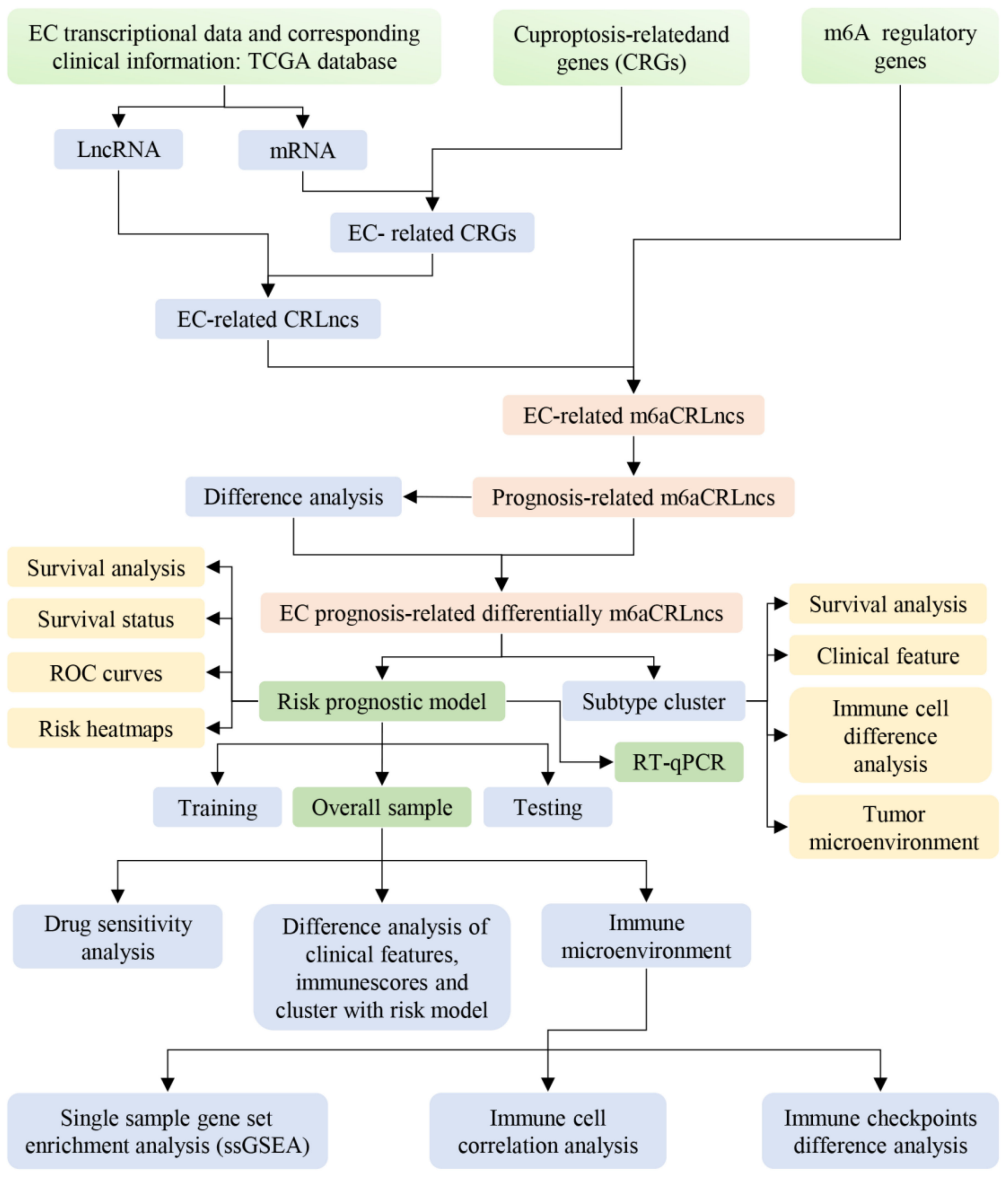
Flow diagram of our study.

To enhance the clarity and comprehensibility of our study, a flowchart was constructed, as illustrated in **Figure 1**.

### EC prognosis-related differentially expressed m6aCRLncs

**Figure 2:**
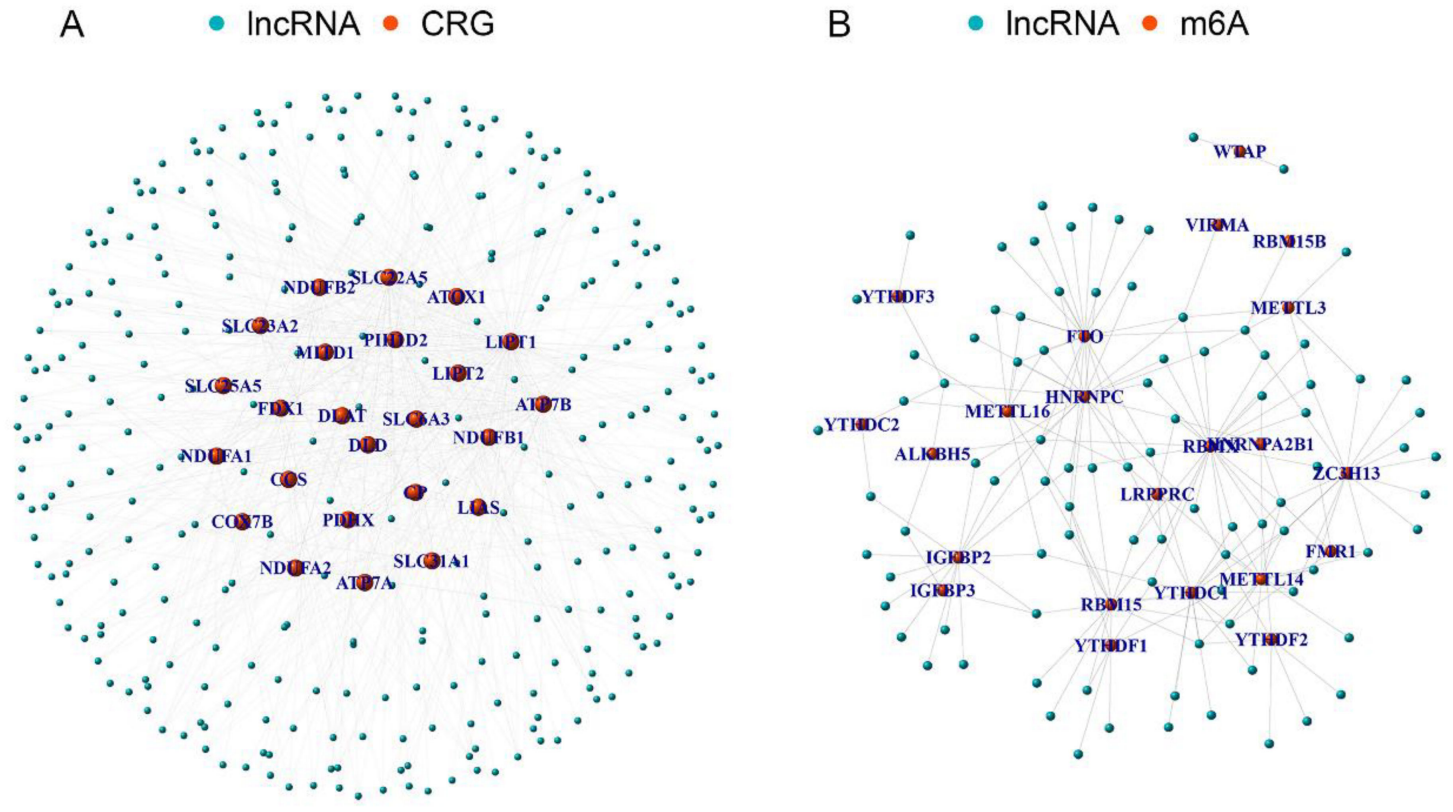
The m6aCRLncs were obtained. **(A)** A total of 335 CRLncs were identified by co-expression analysis of 25 CRGs and 3719 lncRNAs. **(B)** A total of 92 m6aCRLncs were identified by co-expression analysis of 335 CRLncs and 23 m6A regulators.

**Figure 3:**
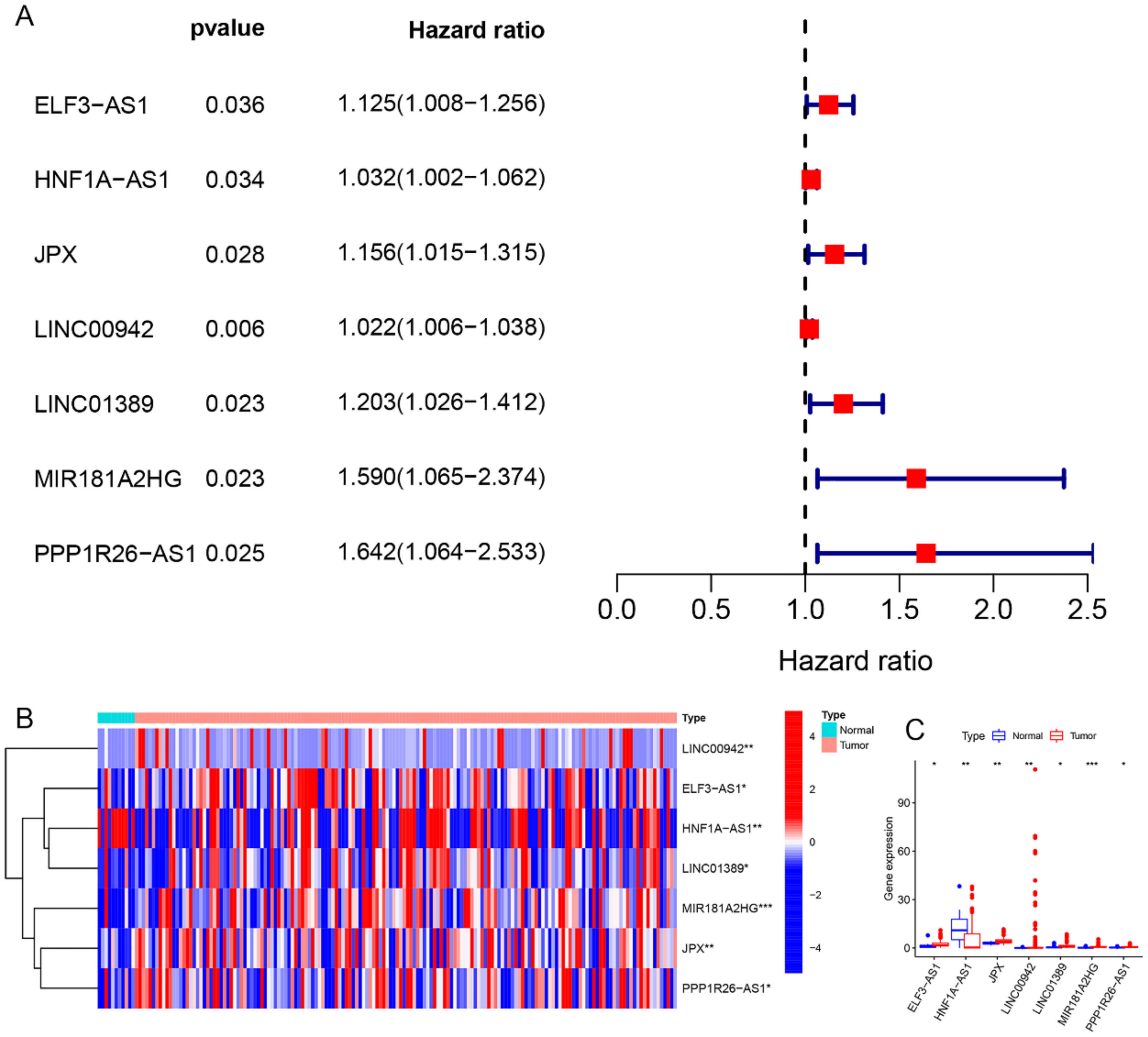
EC prognosis-related differentially expressed m6aCRLncs. **(A)** 7 m6aCRLncs were demonstrated by univariate Cox regression analysis as having prognostic significance. **(B)** Difference heatmap of m6aCRLncs associated with EC prognosis. **(C)** Difference boxplot of m6aCRLncs associated with EC prognosis. (* *P*< 0.05, ** *P<* 0.01, *** *P*< 0.001).

The 25 CRGs were cross-referenced with the gene expression data from the EC microarray, resulting in the identification of 25 EC-related CRGs. Through co-expression analysis of these CRGs and lncRNAs in EC RNA-seq data, a total of 335 EC-related CRLncs were identified (**Figure 2A**). Further co-expression analysis between the EC-related CRLncs and 23 m6A regulators led to the identification of 92 EC-related m6aCRLncs (**Figure 2B**). Subsequently, seven EC prognosis-related m6aCRLncs were identified through single-variable Cox regression analysis (**Figure 3A**). These seven m6aCRLncs exhibited differential expression between normal and EC samples (**Figures 3B, C**).

### Subtype cluster analysis of EC prognosis-related differentially m6aCRLncs

**Figure 4:**
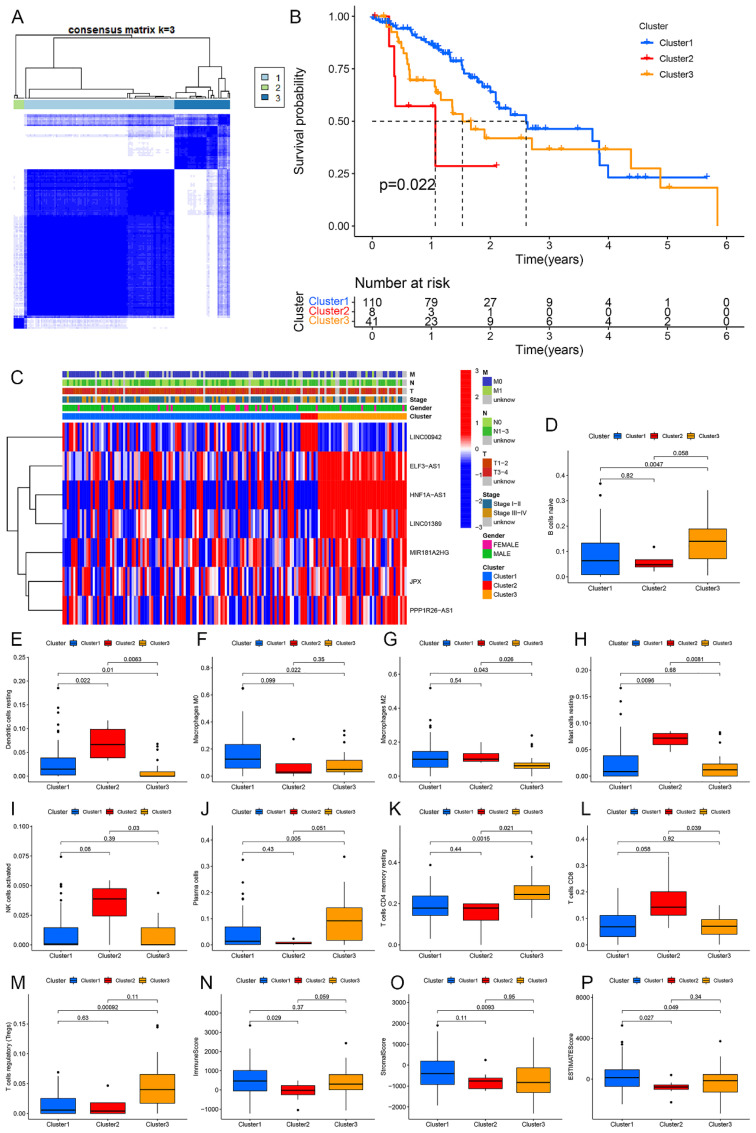
Subtype cluster analysis of m6aCRLncs. **(A)** At K = 3, the classification was the most reliable. **(B)** Survival analysis of different subtypes of EC patients. **(C)** Clinical characteristics analysis of EC patients with different subtypes. **(D-M)** Immune cells differences analysis of EC patients with different subtypes. (**N-P)** Immune microenvironment differences analysis of EC patients with different subtypes.

To assess whether the seven m6aCRLncs could be used to cluster EC patients, a subtype clustering analysis was conducted. The optimal clustering stability was achieved when K = 3 (**Figure 4A**), resulting in the classification of 159 EC patients into three distinct clusters: Cluster 1 (N = 110), Cluster 2 (N = 8), and Cluster 3 (N = 41). Survival analysis revealed that Cluster 2 exhibited the poorest prognosis among the three groups, with a statistically significant difference in survival outcomes (P = 0.022) (**Figure 4B**). No significant differences were observed in clinical features among the different EC subtypes (**Figure 4C**). Further differential analysis of 22 immune cells and subtypes identified notable variations, with resting dendritic cells showing differences among all three clusters. Additionally, differences in immune cell populations, including naive B cells, resting macrophages (M0), M2 macrophages, resting mast cells, activated NK cells, plasma cells, resting memory CD4+ T cells, CD8+ T cells, and regulatory T cells (Tregs), were observed between one or two of the EC subtypes (**Figures 4D-M**). Finally, differential analysis of the tumor microenvironment revealed significant variations in immune scores, stromal scores, and ESTIMATE scores between one or two of the EC subtypes (**Figures 4N-P**).

### Construction of risk prognostic model

**Figure 5:**
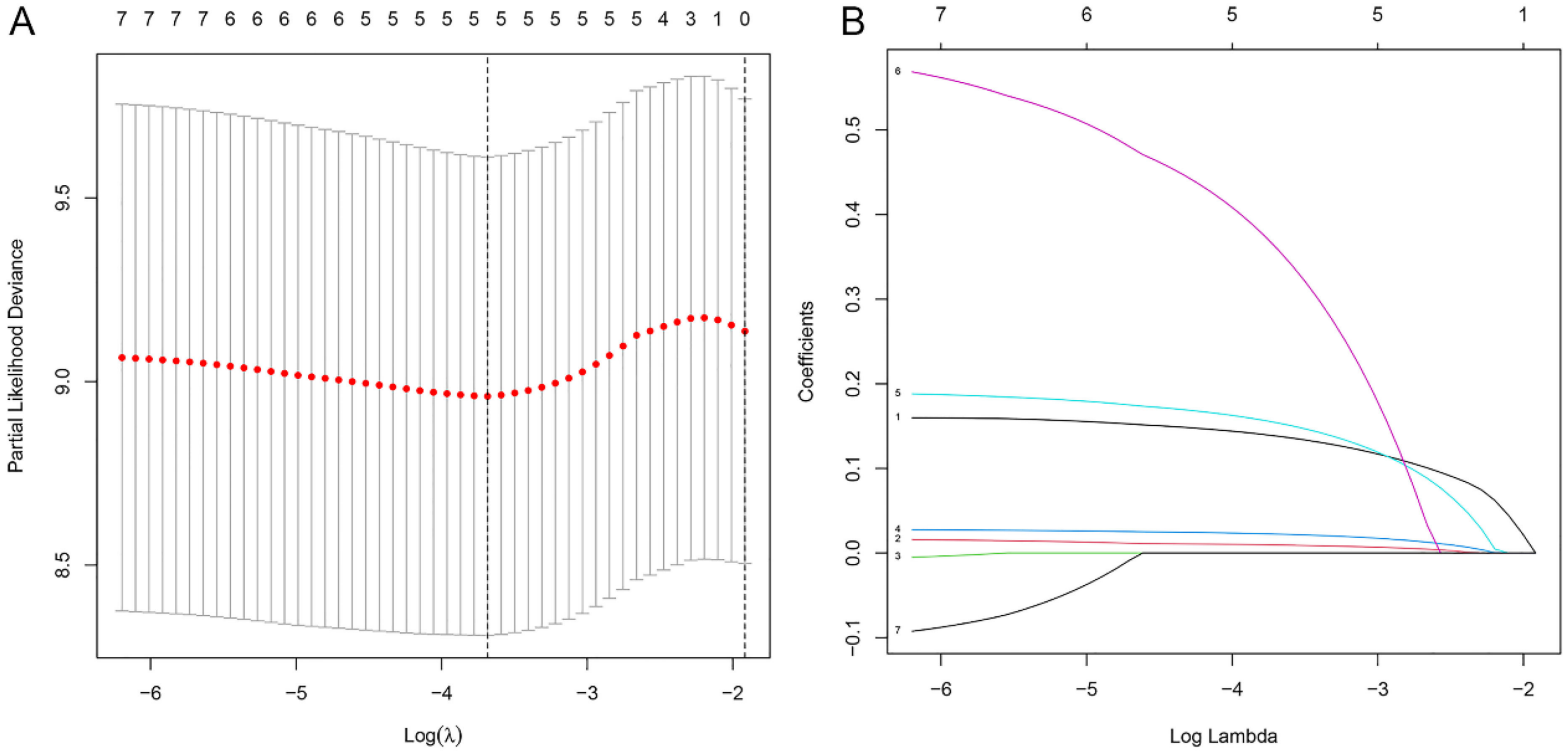
Construction of m6aCRLncs risk prognostic model. **(A)** Five m6aCRLncs’ LASSO coefficient profiles. **(B)** Cross-validation to fine-tune the LASSO model’s parameter selection.

In the preceding univariate Cox regression analysis, seven differentially expressed m6aCRLncs were identified as being prognostically relevant to EC. Subsequently, LASSO regression analysis revealed that five m6aCRLncs constituted the optimal number to include in the model, as determined by the most favorable penalty parameter (λ) value (**Figures 5A, B**). Based on this model, the overall sample cohort was stratified into high-risk (N = 72) and low risk (N = 87) groups. Similarly, the training cohort was divided into high-risk (N = 40) and low-risk (N = 40) groups, while the testing cohort was separated into high-risk (N = 32) and low-risk (N = 47) groups.

### Risk prognosis models forecast the prognosis of EC patients

**Figure 6:**
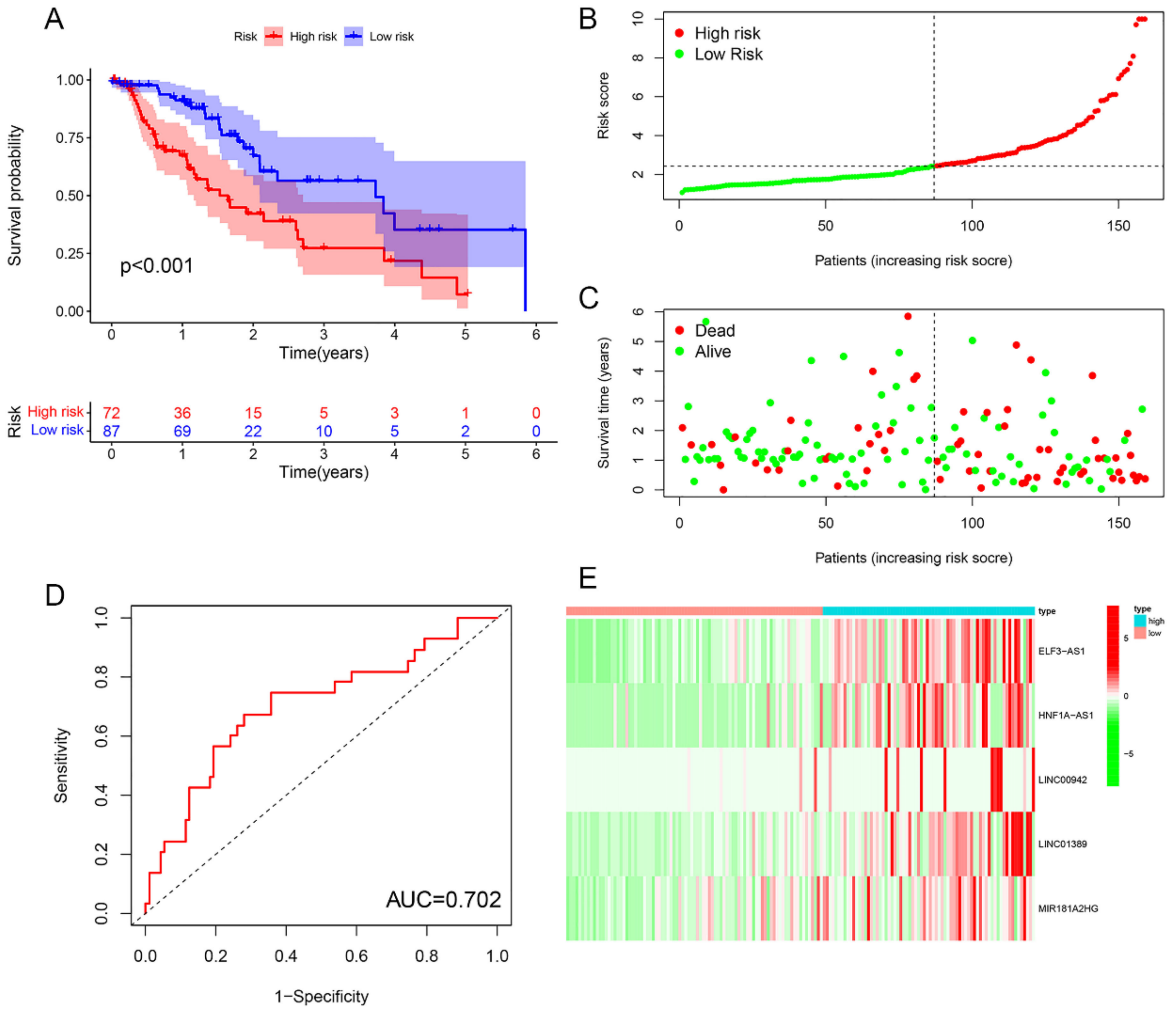
Overall sample cohort. **(A)** Survival curve. **(B, C)** Survival status map. **(D)** ROC curve. **(E)** Risk heatmap.

**Figure 7:**
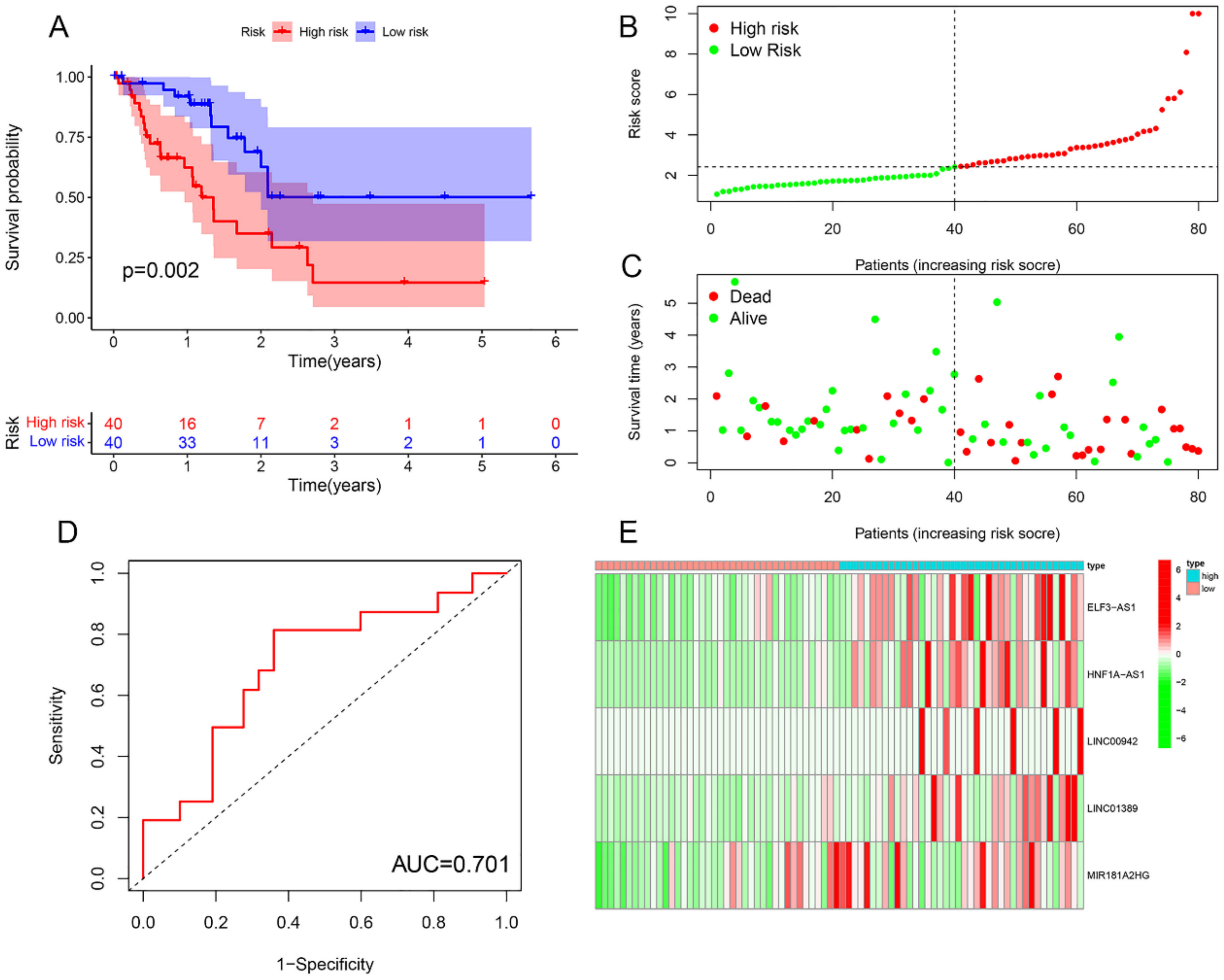
Training cohort. **(A)** Survival curve. **(B, C)** Survival status map. **(D)** ROC curve. **(E)** Risk heatmap.

**Figure 8:**
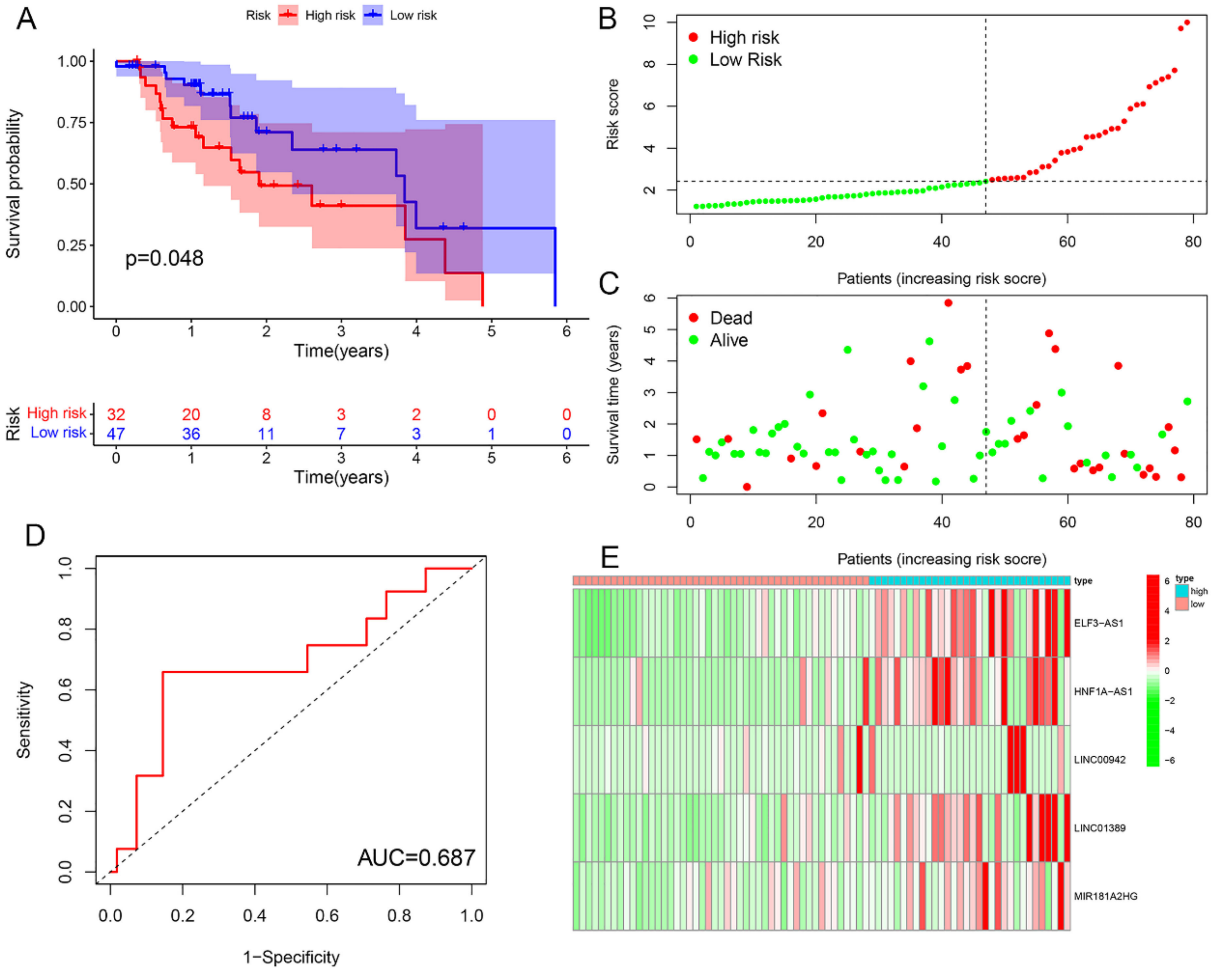
Testing cohort. **(A)** Survival curve. **(B, C)** Survival status map. **(D)** ROC curve. **(E)** Risk heatmap.

Survival analysis revealed statistically significant differences in survival outcomes between the high- and low-risk groups, with patients in the low-risk group exhibiting better survival rates compared to those in the high-risk group. This pattern was observed across the overall sample group (P< 0.001), the training group (P = 0.002), and the testing group (P = 0.048) (**Figures 6A**, **7A**, **8A**). Mortality rates among EC patients progressively increased from the low-risk group to the high-risk group, as shown in the survival status plots for the overall sample, training, and testing groups (**Figures 6B, C**, **7B, C**, **8B, C**). This trend underscores that higher risk scores are associated with poorer survival outcomes. ROC curve analysis demonstrated that the 1-year area under the curve (AUC) values for the overall sample group, training group, and testing group were 0.702, 0.701, and 0.687, respectively (**Figures 6D**, **7D**, **8D**). Furthermore, the expression levels of five m6aCRLncs, namely ELF3-AS1, HNF1A-AS1, LINC00942, LINC01389, and MIR181A2HG, were identified as high-risk factors for EC, with their expression levels increasing progressively from the low-risk group to the high-risk group (**Figures 6E**, **7E**, **8E**).

### Difference analysis of clinical features, immunescores and cluster with risk model

**Figure 9:**
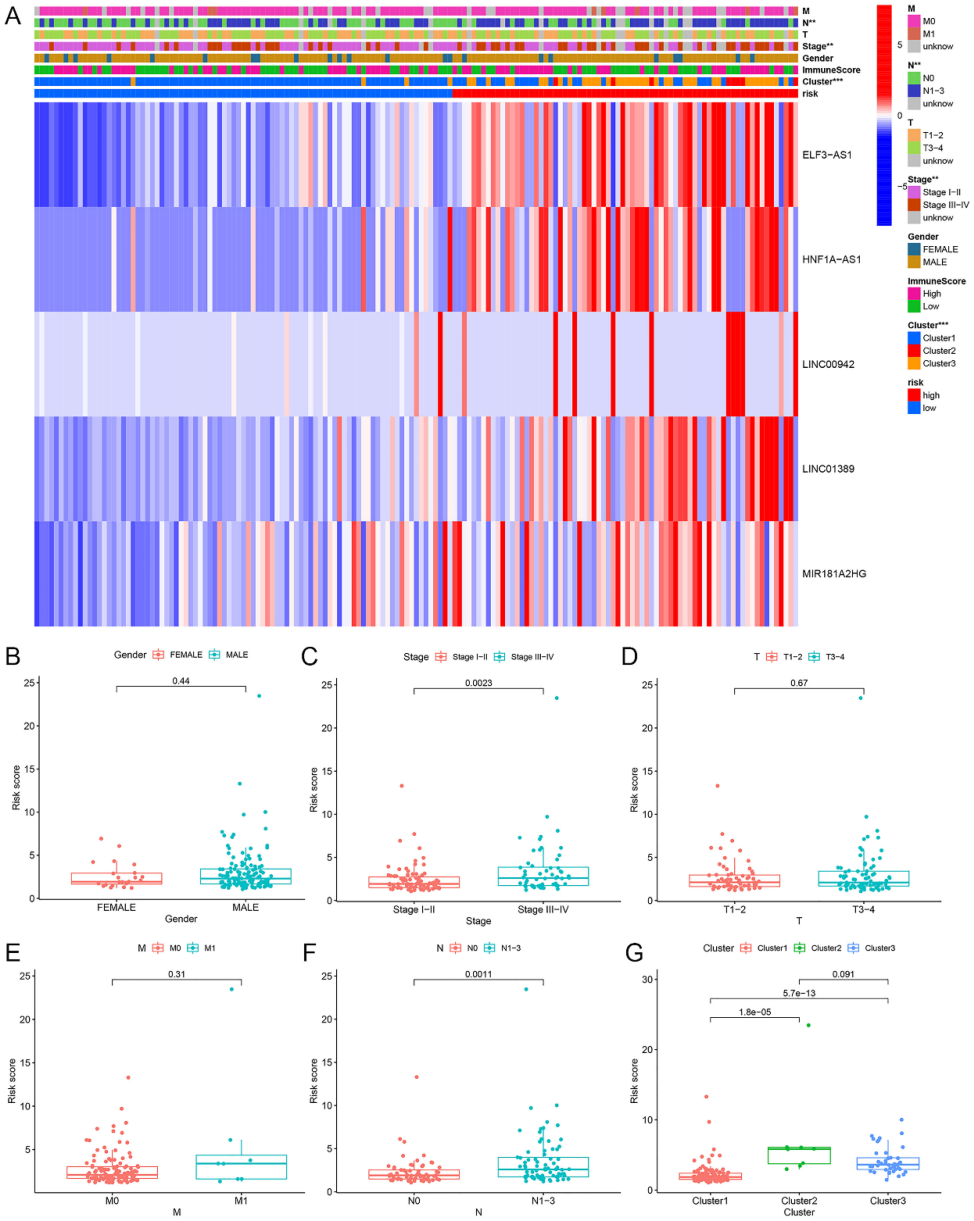
Relationship between riskscore and cluster, immune score, and clinical features (gender, clinical stage, T, N, M) in high- and low-risk groups within the overall sample cohort. **(A)** Heatmap. **(B-G)** Boxplot.

1?>Heatmaps and boxplots were generated to analyze the associations between clusters, immunescores, clinical features (including gender, clinical stage, T, N, and M), and risk scores in both high- and low-risk groups within the overall sample cohort. The results indicated significant differences in cluster, clinical stage, and N stage between the high-risk and low-risk groups (**Figure 9**).

### Risk prognosis model guide the immune microenvironment of EC patients

**Figure 10:**
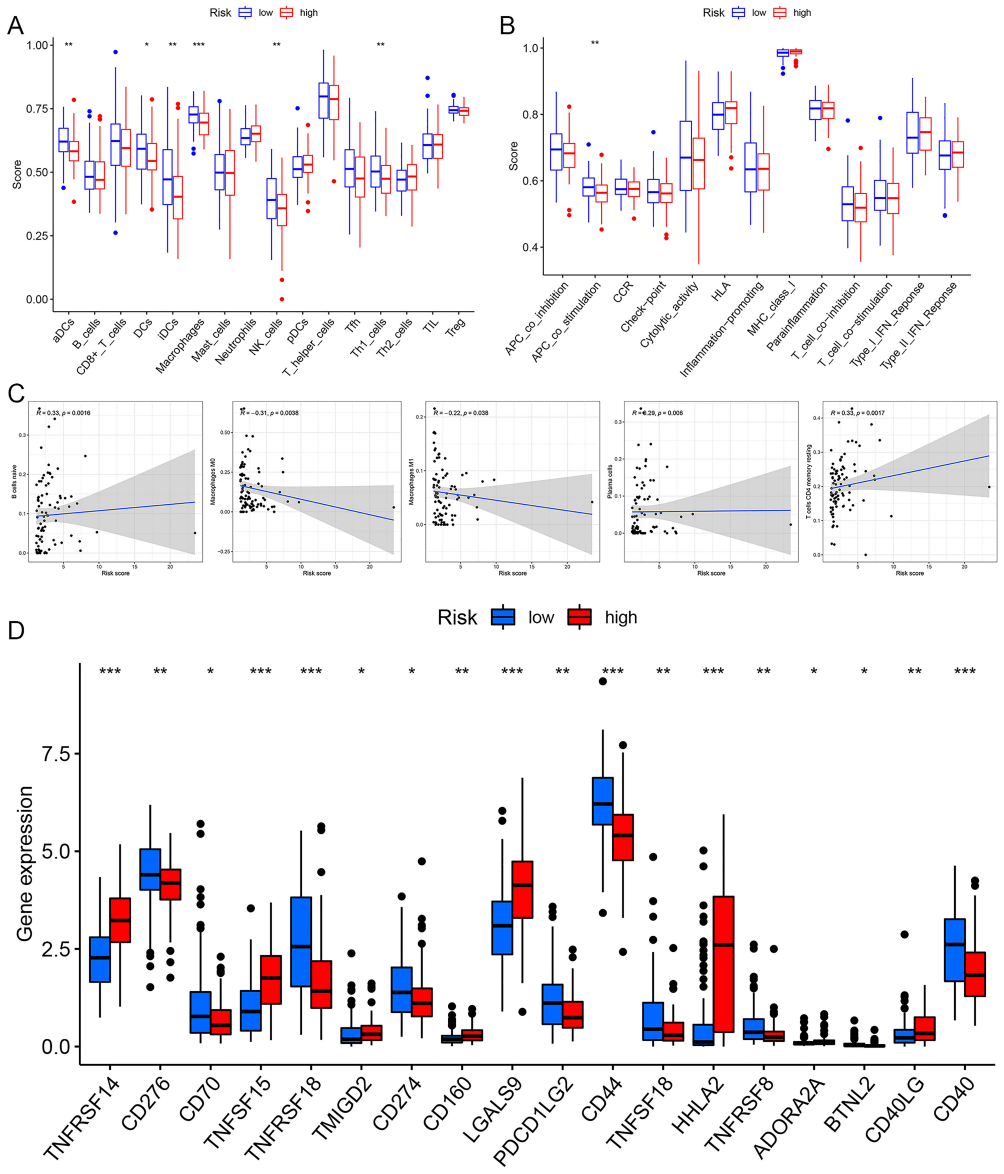
The immune correlation analysis of risk prognosis model. **(A)** Immune cell differential analysis. **(B)** Immune function differential analysis. **(C)** The correlation analysis correlation between riskscore and immune cell. **(D)** Immune checkpoint analysis. *p< 0.05, **p< 0.01, ***p< 0.001.

Differential analysis of immune cell populations revealed a significant reduction in the levels of aDCs, DCs, iDCs, macrophages, NK cells, and Th1 cells in the high-risk group (**Figure 10A**). Immune function analysis indicated a notable downregulation of APC co-stimulation in the high-risk group (**Figure 10B**). Immune correlation analysis identified a positive association between risk score and the levels of naive B cells, resting CD4 T cells, and plasma cells, with higher content of these immune cells correlating with an increased risk of EC. Conversely, a negative correlation was observed between risk score and macrophages M0 and M1, with higher content of these cell types linked to a reduced risk of EC (**Figure 10C**). Immune checkpoint analysis showed that patients in the high-risk group exhibited upregulation of TNFRSF14, TNFSF15, CD160, LGALS9, HHLA2, and CD40LG, while the expression levels of CD276, TNFRSF18, PDCD1LG2, CD44, TNFSF18, TNFRSF8, and CD40 were downregulated in the high-risk patients (**Figure 10D**).

### Potential therapeutic drugs for EC patients

**Figure 11:**
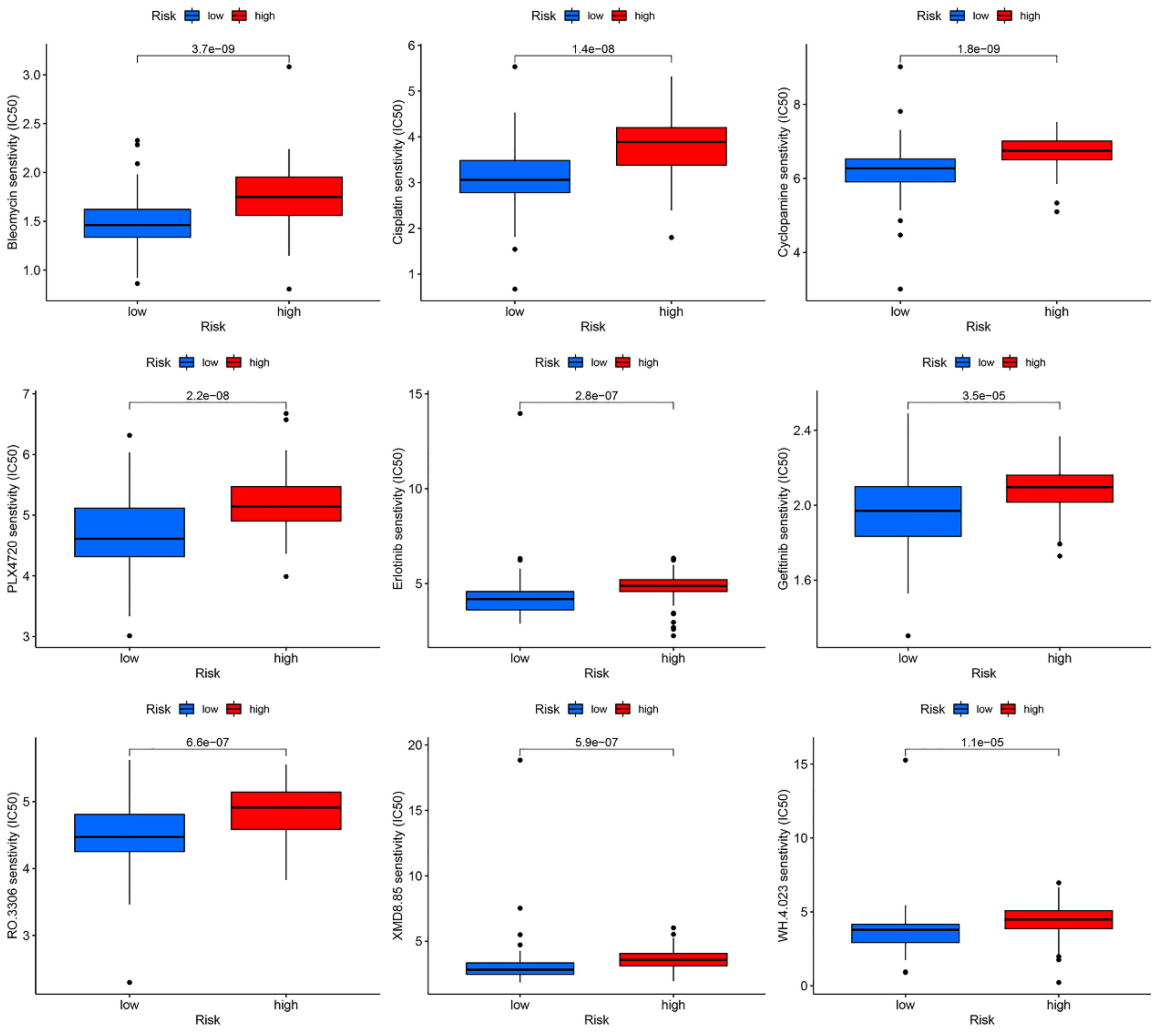
Relationship between risk prognostic model and sensitivity drugs in EC patients.

A drug sensitivity analysis revealed that Bleomycin, Cisplatin, Cyclopamine, PLX4720, Erlotinib, Gefitinib, RO.3306, XMD8.85, and WH.4.023 exhibited marked sensitivity in both the high- and low-risk groups. Notably, patients in the low-risk group showed significantly greater responsiveness to these nine drugs compared to those in the high-risk group (**Figure 11**).

### Validation of the expression of m6aCRLncs in EC

**Figure 12:**
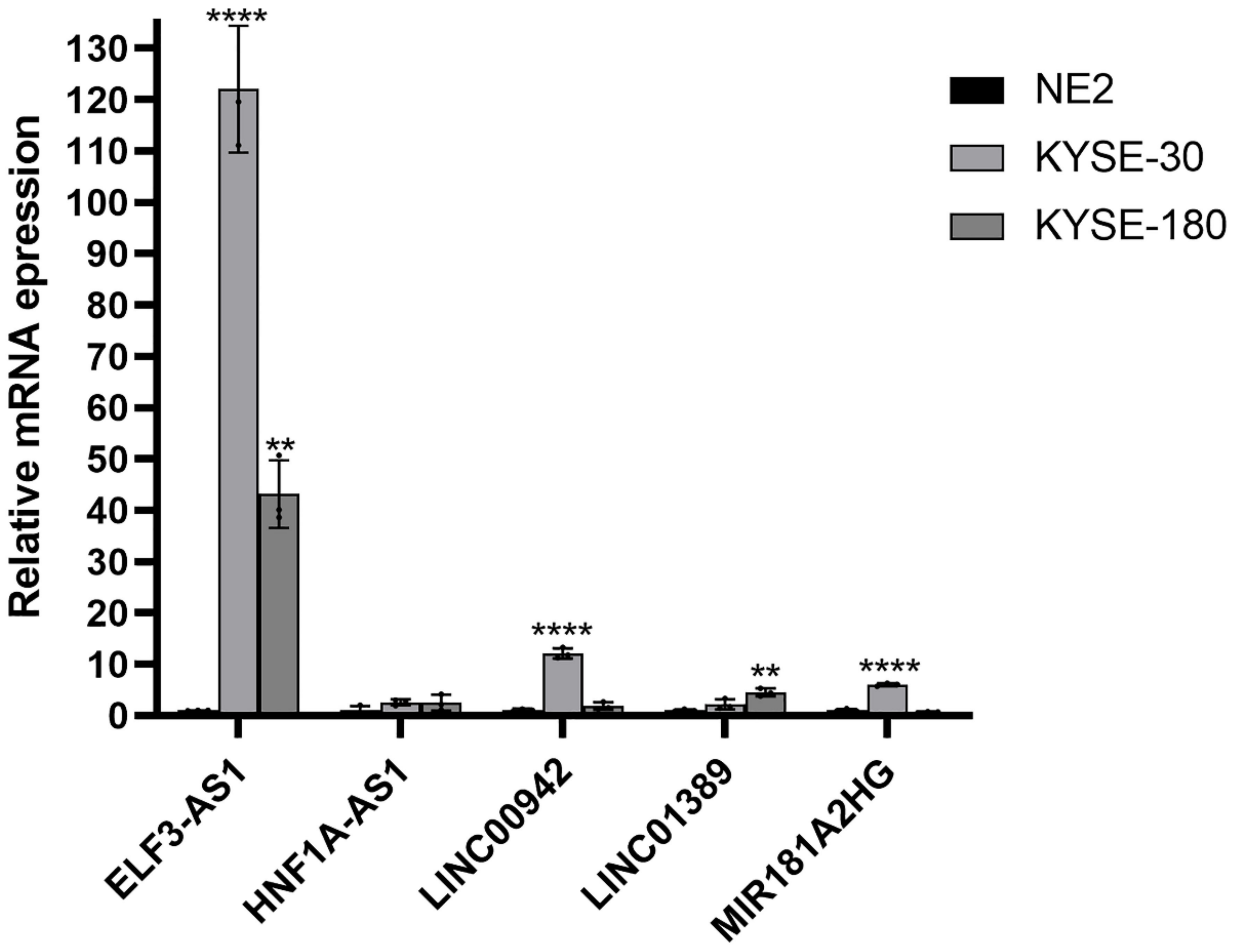
Validation of the mRNA expression level of m6aCRLncs in EC cell lines. **p< 0.01, ****p< 0.0001, each experiment was repeated three times.

To further assess the expression of m6aCRLncs in EC, two EC cell lines, KYSE-30 and KYSE-180, were selected for evaluation of mRNA expression levels, with normal esophageal epithelial cells (NE-2) serving as the control group. The results revealed a significant upregulation of ELF3-AS1 mRNA expression in both KYSE-30 and KYSE-180 cell lines compared to NE-2. Furthermore, mRNA expression levels of LINC01389 and MIR181A2HG were markedly elevated in the KYSE-180 cell line relative to the control. Additionally, LINC00942 mRNA expression was notably higher in the KYSE-30 cell line compared to NE-2 (**Figure 12**).

## Discussion

This study developed a novel prognostic risk model for EC, leveraging five m6aCRLncs (ELF3-AS1, HNF1A-AS1, LINC00942, LINC01389, and MIR181A2HG) identified as high-risk factors for EC patients. Comprehensive validation demonstrated that the model effectively predicts patient survival outcomes and provides insights into the immune microenvironment of EC. Additionally, subtype clustering and correlation analyses with clinical features revealed significant differences in cluster composition, clinical stage, and N stage between high- and low-risk groups. Immune profiling further highlighted disparities between these groups, with naive B cells, resting CD4 T cells, and plasma cells positively correlating with risk scores, whereas macrophages M0 and M1 displayed negative correlations. Differential expression of immune checkpoint-related genes, including TNFRSF14, TNFSF15, TNFRSF18, LGALS9, CD44, HHLA2, and CD40, provided additional mechanistic insights. Lastly, drug sensitivity analysis identified nine therapeutic agents with potential efficacy for EC patients, including Bleomycin, Cisplatin, Cyclopamine, PLX4720, Erlotinib, Gefitinib, RO.3306, XMD8.85, and WH.4.023, offering promising avenues for personalized treatment strategies.

In this study, we identified seven m6aCRLncs significantly associated with the prognosis of EC through univariate Cox regression analysis. These m6aCRLncs included ELF3-AS1, HNF1A-AS1, JPX, LINC00942, LINC01389, MIR181A2HG, and PPP1R26-AS1. As illustrated in **Figure 3A**, all seven m6aCRLncs exhibited hazard ratio (HR) greater than 1, indicating their classification as high-risk m6aCRLncs implicated in the progression and pathogenesis of EC. Differential expression analysis was subsequently conducted to compare these m6aCRLncs between the control group (comprising 11 normal samples) and the tumor group (comprising 159 EC patients), as shown in **Figures 3B, C**. The results demonstrated that ELF3-AS1, JPX, LINC00942, LINC01389, MIR181A2HG, and PPP1R26-AS1 were significantly upregulated in EC patients, whereas HNF1A-AS1 showed reduced expression in the EC group. To further refine prognostic relevance, a risk-prognosis model was developed, which identified five key m6aCRLncs (ELF3-AS1, HNF1A-AS1, LINC00942, LINC01389, and MIR181A2HG) as significant contributors to patient stratification. As depicted in **Figures 6E**, **7E**, and **8E**, these five m6aCRLncs exhibited higher expression levels in the high-risk EC patient group compared to the low-risk group. Notably, four of these m6aCRLncs (ELF3-AS1, LINC00942, LINC01389, and MIR181A2HG) were consistently overexpressed in both the overall EC cohort and the high-risk subgroup, suggesting their critical roles in EC pathobiology. The findings were further validated through PCR analysis, which confirmed the elevated expression of ELF3-AS1, LINC00942, LINC01389, and MIR181A2HG in EC patients compared to controls, as shown in **Figure 12**. These results collectively underscore the potential of these m6aCRLncs as biomarkers for prognosis and as targets for therapeutic intervention in EC.

In contrast to the four m6aCRLncs (ELF3-AS1, LINC00942, LINC01389, and MIR181A2HG), HNF1A-AS1 exhibits a unique expression pattern in EC patients. Although HNF1A-AS1 is more highly expressed in the high-risk group compared to the low-risk group of EC patients, it shows decreased expression in the tumor group (comprising 159 EC samples) compared to the control group (11 normal samples). It is important to note that the high- and low-risk groups within the risk prognosis model consist solely of EC patients, and the comparisons do not involve normal controls. Consequently, the elevated expression of HNF1A-AS1 in high-risk EC patients relative to the low-risk group does not imply that its expression is generally higher in EC patients when compared to healthy individuals. Differential expression analysis revealed lower expression levels of HNF1A-AS1 in EC patients relative to normal controls, although its expression remained significantly different between the high-risk and low-risk subgroups, with higher levels observed in high-risk patients. However, the PCR validation results did not show statistically significant differences in HNF1A-AS1 expression between EC patients and controls. While HNF1A-AS1 appeared to be moderately overexpressed in EC patients, as depicted in **Figure 12**, this observation lacked statistical significance. Such discrepancies between bioinformatics predictions and PCR validation are not uncommon and reflect the inherent limitations of computational analysis, particularly when combined with experimental methods. Importantly, four of the five m6aCRLncs included in the risk-prognosis model (ELF3-AS1, LINC00942, LINC01389, and MIR181A2HG) were successfully validated by PCR, which sufficiently supports the overall findings and biological significance of the model. The inconsistent results for HNF1A-AS1 may be influenced by the disparity in sample sizes between the two groups analyzed. Specifically, the differential expression analysis compared 11 normal samples with 159 EC samples, and the imbalanced sample sizes may have contributed to variations in the statistical outcomes for HNF1A-AS1. Unfortunately, the current dataset did not allow for analyses using a more balanced or larger sample size, representing a limitation of this study. Addressing this limitation in future research through studies with larger and more evenly distributed sample sizes will be crucial to better understand the expression patterns and biological roles of HNF1A-AS1 in EC prognosis and pathogenesis.

This study identified ELF3-AS1, LINC00942, LINC01389 and MIR181A2HG as being significantly associated with tumor prognosis of EC patients, thereby reinforcing the validity of the findings. The prognostic value of HNF1A-AS1 in EC patients needs further study. ELF3-AS1 has been strongly linked to the prognosis of glioma and hepatocellular carcinoma (37–39) and has been shown to accelerate gastric cancer progression through binding to hnRNPK (40). LINC00942 has been linked to prognosis and immune responses in hepatocellular and bladder cancers (41–45) and promotes METTL14-mediated m6A methylation in breast cancer (46). Although the role of LINC01389 in tumor prognosis remains uncertain, it has been shown to participate in the epithelial-mesenchymal transition in stomach cancer (47). MIR181A2HG is associated with prognostic prediction and immunotherapy response in bladder cancer (48) and serves as a diagnostic marker for thyroid cancer (49). HNF1A-AS1 is implicated in osteosarcoma prognosis and tumorigenesis (50) and plays roles in the progression of gastric cancer and glioblastoma (50–52). Specifically, it acts as a competitive endogenous RNA in gastric cancer by sponging miR-30b-3p (34) and, when regulated by HNF1α, mitigates hepatocellular carcinoma malignancy by enhancing SHP-1 phosphatase activity (53). Importantly, HNF1A-AS1 is also involved in the development of EC. Studies reveal its role in regulating proliferation and migration in esophageal adenocarcinoma (EAC) cells (54) and promoting growth and metastasis in esophageal squamous cell carcinoma (ESCC) by sponging miR-214 to upregulate SOX-4 expression (55). These findings highlight the multifaceted roles of these m6aCRLncs in cancer progression and their potential as biomarkers or therapeutic targets.

Tumor immunotherapy represents a promising and innovative therapeutic strategy for EC (6). EC cells are characterized by a rich repertoire of tumor antigens, yet they have developed sophisticated mechanisms to evade anti-tumor immune responses. These mechanisms include activation of immune checkpoints, secretion of immunosuppressive factors, and negative regulation of immune cell activity. The immune landscape within the tumor microenvironment significantly influences cancer progression, patient survival, and treatment efficacy (6). Immune checkpoints play a pivotal role in maintaining self-tolerance and preventing the onset of inflammatory disorders. However, cancer cells can exploit these pathways to induce T-cell exhaustion and impair immune function. In recent years, immunotherapy targeting immune checkpoints has seen remarkable advancements, providing new hope for improving clinical outcomes in EC (56).

The genetic variant rs2234167 within the TNFRSF14 locus has not been associated with the risk of ESCC (57). However, this study revealed that TNFRSF14 expression was upregulated in high-risk EC patients, suggesting its potential involvement in the risk of EC. Additionally, CD44 was found to be downregulated in high-risk EC patients, indicating its possible role in the immunotherapy of EC. Previous studies have identified CD44 as a novel biomarker for EC patients undergoing neoadjuvant chemoradiotherapy and highlighted its utility, in combination with HER2, in enhancing the predictive accuracy of 18F-FDG PET-based clinico-radiomic models for treatment response (58, 59). The immune checkpoint molecule HHLA2 has been shown to predict survival and immune characteristics in patients with ESCC (60). This study reinforces the prognostic and immunotherapeutic potential of HHLA2 in EC. Furthermore, the expression of CD40 in human ESCC has been linked to tumor progression and lymph node metastasis (61). Currently, limited data exists regarding the roles of TNFSF15, TNFRSF18, and LGALS9 in EC. This study identifies these molecules as being associated with the prognosis and immune microenvironment of EC, providing new directions for future research on EC prognosis and immunotherapeutic strategies.

Bleomycin and Cisplatin are established chemotherapeutic agents for EC (62, 63). TNFAIP8 has been implicated in promoting Cisplatin resistance via interaction with TAF-Iα, thereby contributing to the malignant progression of EC (64). Cyclopamine exerts its anti-tumor effects by inhibiting glioma-associated oncogene protein-1, a key marker of EC progression, effectively suppressing the growth of EC cells (65). Furthermore, treatment with KAAD-Cyclopamine or neutralizing antibodies targeting Shh has been shown to reduce EC cell proliferation and induce apoptosis (66). For patients with ESCC who are intolerant to chemoradiotherapy, Erlotinib in combination with radiotherapy has demonstrated therapeutic efficacy (67). Similarly, Gefitinib has been shown to enhance survival and improve quality of life in advanced-stage EC patients who have failed first-line chemotherapy (68). Among the drugs identified as sensitive in this study, several are known to exhibit therapeutic potential in EC, underscoring the robustness of the study’s findings. The roles of PLX4720, RO.3306, XMD8.85, and WH.4.023 in EC have not been well-documented. These agents may represent promising candidates for further exploration as potential therapeutic options for EC.

This study, while offering meaningful insights, is not without its limitations. First, the relatively small sample size of tumor specimens, ​which may constrain the statistical power and generalizability of the findings. A larger, more diverse cohort is essential in future research to ensure the robustness and reproducibility of the conclusions. Expanding the sample size would not only strengthen the statistical validity of the prognostic model but also enhance its applicability across varied patient populations. Furthermore, a larger cohort would provide an opportunity to validate the identified biomarkers in independent datasets, thereby increasing the reliability of the proposed risk stratification framework for clinical implementation. Second, although this research identifies key m6aCRLncs with prognostic and immune-regulatory roles in EC, their underlying biological mechanisms remain largely unexplored. Comprehensive functional analyses are required to elucidate how these m6aCRLncs contribute to tumor progression, immune microenvironment modulation, and therapy resistance. This would provide deeper insights into their potential as biomarkers or therapeutic targets. Addressing these limitations in subsequent studies will be crucial to confirming the translational potential of the findings and further advancing the field of EC research.

## Conclusion

In conclusion, this study successfully established a novel prognostic model for EC based on five m6aCRLncs, offering a comprehensive approach to risk stratification. These five m6aCRLncs demonstrated significant potential in predicting immune efficacy and drug sensitivity, as evidenced by analyses of the tumor microenvironment, immune correlations, and drug response patterns. The integration of these biomarkers into a prognostic framework not only provides valuable insights into the immune landscape of EC but also highlights their utility in identifying potential therapeutic options. The findings of this research hold promising implications for predicting patient survival and optimizing clinical management strategies, and lay a theoretical foundation for more personalized and effective treatment protocols for EC patients.

## Data Availability

Publicly available datasets were analyzed in this study. This data can be found here: https://portal.gdc.cancer.gov/analysis_page?app=Downloads.
