# Supplementary material for: Subtype cluster analysis unveiled the correlation between m6A- and cuproptosis-related lncRNAs and the prognosis, immune microenvironment, and treatment sensitivity of esophageal cancer
Source: Front Immunol. 2025 Feb 17;16:1539630. doi: 10.3389/fimmu.2025.1539630 (PMC11872909; doi:10.3389/fimmu.2025.1539630)
Supplement: Supplementary file 3 [file Table1.docx]

**1. cuproptosis-related genes**

#if (!requireNamespace("BiocManager", quietly = TRUE))

# install.packages("BiocManager")

#BiocManager::install("limma")

library(limma)

setwd("")

rt=read.table("mRNA.txt",sep="\t",header=T,check.names=F)

rt=as.matrix(rt)

rownames(rt)=rt[,1]

exp=rt[,2:ncol(rt)]

dimnames=list(rownames(exp),colnames(exp))

data=matrix(as.numeric(as.matrix(exp)),nrow=nrow(exp),dimnames=dimnames)

data=avereps(data)

data=data[rowMeans(data)>0,]

gene=read.table("gene.txt", header=F, check.names=F, sep="\t")

sameGene=intersect(as.vector(gene[,1]),rownames(data))

geneExp=data[sameGene,]

out=rbind(ID=colnames(geneExp),geneExp)

write.table(out,file="CRGexp.txt",sep="\t",quote=F,col.names=F)

**2. cuproptosis-related lncRNAs**

#if (!requireNamespace("BiocManager", quietly = TRUE))

# install.packages("BiocManager")

#BiocManager::install("limma")

library(limma)

setwd("")

corFilter=0.3

pvalueFilter=0.001

rt = read.table("lncRNA.txt",header=T,sep="\t",check.names=F)

rt=as.matrix(rt)

rownames(rt)=rt[,1]

exp=rt[,2:ncol(rt)]

dimnames=list(rownames(exp),colnames(exp))

lncRNA=matrix(as.numeric(as.matrix(exp)),nrow=nrow(exp),dimnames=dimnames)

lncRNA=avereps(lncRNA)

lncRNA=lncRNA[rowMeans(lncRNA)>0.5,]

group=sapply(strsplit(colnames(lncRNA),"\\-"),"[",4)

group=sapply(strsplit(group,""),"[",1)

group=gsub("2","1",group)

lncRNA=lncRNA[,group==0]

rt = read.table("CRGexp.txt",header=T,sep="\t",check.names=F)

rt=as.matrix(rt)

rownames(rt)=rt[,1]

exp=rt[,2:ncol(rt)]

dimnames=list(rownames(exp),colnames(exp))

CRGgene=matrix(as.numeric(as.matrix(exp)),nrow=nrow(exp),dimnames=dimnames)

CRGgene=avereps(CRGgene)

CRGgene=CRGgene[rowMeans(CRGgene)>0.5,]

group=sapply(strsplit(colnames(CRGgene),"\\-"),"[",4)

group=sapply(strsplit(group,""),"[",1)

group=gsub("2","1",group)

CRGgene=CRGgene[,group==0]

outTab=data.frame()

for(i in row.names(lncRNA)){

if(sd(lncRNA[i,])>0.5){

for(j in row.names(CRGgene)){

x=as.numeric(lncRNA[i,])

y=as.numeric(CRGgene[j,])

corT=cor.test(x,y)

cor=corT$estimate

pvalue=corT$p.value

if((abs(cor)>corFilter) & (pvalue<pvalueFilter)){

outTab=rbind(outTab,cbind(CRGgene=j,lncRNA=i,cor,pvalue))

}

}

}

}

write.table(file="corResult.txt",outTab,sep="\t",quote=F,row.names=F)

CRGlncRNA=unique(as.vector(outTab[,"lncRNA"]))

CRGlncRNAexp=lncRNA[CRGlncRNA,]

CRGlncRNAexp=rbind(ID=colnames(CRGlncRNAexp),CRGlncRNAexp)

write.table(CRGlncRNAexp,file="CRGlncRNAexp.txt",sep="\t",quote=F,col.names=F)

**3. The expression of m6A gene was extracted**

#if (!requireNamespace("BiocManager", quietly = TRUE))

# install.packages("BiocManager")

#BiocManager::install("limma")

library(limma)

expFile="symbol.txt"

geneFile="gene.txt"

setwd("")

rt=read.table(expFile, header=T, sep="\t", check.names=F)

rt=as.matrix(rt)

rownames(rt)=rt[,1]

exp=rt[,2:ncol(rt)]

dimnames=list(rownames(exp),colnames(exp))

data=matrix(as.numeric(as.matrix(exp)),nrow=nrow(exp),dimnames=dimnames)

data=avereps(data)

data=data[rowMeans(data)>0,]

gene=read.table(geneFile, header=T, check.names=F, sep="\t")

sameGene=intersect(as.vector(gene[,1]), rownames(data))

geneExp=data[sameGene,]

out=rbind(ID=colnames(geneExp),geneExp)

write.table(out,file="m6aGeneExp.txt",sep="\t",quote=F,col.names=F)

**4.** **Co-expression network**

#if (!requireNamespace("BiocManager", quietly = TRUE))

# install.packages("BiocManager")

#BiocManager::install("limma")

library(limma)

corFilter=0.3

pvalueFilter=0.001

setwd("")

rt=read.table("lncRNA.txt", header=T, sep="\t", check.names=F)

rt=as.matrix(rt)

rownames(rt)=rt[,1]

exp=rt[,2:ncol(rt)]

dimnames=list(rownames(exp),colnames(exp))

data=matrix(as.numeric(as.matrix(exp)),nrow=nrow(exp),dimnames=dimnames)

data=avereps(data)

data=data[rowMeans(data)>0.1,]

group=sapply(strsplit(colnames(data),"\\-"),"[",4)

group=sapply(strsplit(group,""), "[", 1)

group=gsub("2","1",group)

lncRNA=data[,group==0]

conNum=length(group[group==1])

treatNum=length(group[group==0])

sampleType=c(rep(1,conNum), rep(2,treatNum))

rt1=read.table("m6aGeneExp.txt", header=T, sep="\t", check.names=F)

rt1=as.matrix(rt1)

rownames(rt1)=rt1[,1]

exp1=rt1[,2:ncol(rt1)]

dimnames1=list(rownames(exp1),colnames(exp1))

m6A=matrix(as.numeric(as.matrix(exp1)), nrow=nrow(exp1), dimnames=dimnames1)

m6A=avereps(m6A)

m6A=m6A[rowMeans(m6A)>0.1,]

group=sapply(strsplit(colnames(m6A),"\\-"),"[",4)

group=sapply(strsplit(group,""),"[",1)

group=gsub("2","1",group)

m6A=m6A[,group==0]

outTab=data.frame()

for(i in row.names(lncRNA)){

if(sd(lncRNA[i,])>0.1){

test=wilcox.test(data[i,] ~ sampleType)

if(test$p.value<0.05){

for(j in row.names(m6A)){

x=as.numeric(lncRNA[i,])

y=as.numeric(m6A[j,])

corT=cor.test(x,y)

cor=corT$estimate

pvalue=corT$p.value

if((cor>corFilter) & (pvalue<pvalueFilter)){

outTab=rbind(outTab,cbind(m6A=j,lncRNA=i,cor,pvalue,Regulation="postive"))

}

if((cor< -corFilter) & (pvalue<pvalueFilter)){

outTab=rbind(outTab,cbind(m6A=j,lncRNA=i,cor,pvalue,Regulation="negative"))

}

}

}

}

}

write.table(file="net.network.txt",outTab,sep="\t",quote=F,row.names=F)

lncNode=data.frame(Node=unique(as.vector(outTab[,"lncRNA"])), Type="lncRNA")

mrnaNode=data.frame(Node=unique(as.vector(outTab[,"m6A"])), Type="m6A")

nodeOut=rbind(lncNode, mrnaNode)

write.table(nodeOut, file="net.node.txt", sep="\t", quote=F, row.names=F)

m6aLncRNA=unique(as.vector(outTab[,"lncRNA"]))

m6aLncRNAexp=data[m6aLncRNA,]

m6aLncRNAexp=rbind(ID=colnames(m6aLncRNAexp), m6aLncRNAexp)

write.table(m6aLncRNAexp,file="m6aLncExp.txt",sep="\t",quote=F,col.names=F)

**5.LncRNA expression and survival data were pooled**

#if (!requireNamespace("BiocManager", quietly = TRUE))

# install.packages("BiocManager")

#BiocManager::install("limma")

library(limma)

lncFile="m6aLncExp.txt"

cliFile="time.txt"

setwd("")

rt=read.table(lncFile, header=T, sep="\t", check.names=F)

rt=as.matrix(rt)

rownames(rt)=rt[,1]

exp=rt[,2:ncol(rt)]

dimnames=list(rownames(exp), colnames(exp))

data=matrix(as.numeric(as.matrix(exp)), nrow=nrow(exp), dimnames=dimnames)

data=avereps(data)

group=sapply(strsplit(colnames(data),"\\-"),"[",4)

group=sapply(strsplit(group,""),"[",1)

group=gsub("2","1",group)

data=data[,group==0]

colnames(data)=gsub("(.*?)\\-(.*?)\\-(.*?)\\-(.*?)\\-.*", "\\1\\-\\2\\-\\3", colnames(data))

data=t(data)

data=avereps(data)

cli=read.table(cliFile,sep="\t",check.names=F,header=T,row.names=1)

sameSample=intersect(row.names(data),row.names(cli))

data=data[sameSample,]

cli=cli[sameSample,]

out=cbind(cli,data)

out=cbind(id=row.names(out),out)

write.table(out,file="expTime.txt",sep="\t",row.names=F,quote=F)

**6.** **Prognostic LncRNA**

#install.packages("survival")

library(survival)

pFilter=0.05

setwd("")

rt=read.table("expTime.txt", header=T, sep="\t", check.names=F, row.names=1)

rt$futime=rt$futime/365

outTab=data.frame()

sigGenes=c("futime","fustat")

for(gene in colnames(rt[,3:ncol(rt)])){

cox=coxph(Surv(futime, fustat) ~ rt[,gene], data = rt)

coxSummary = summary(cox)

coxP=coxSummary$coefficients[,"Pr(>|z|)"]

if(coxP<pFilter){

sigGenes=c(sigGenes,gene)

outTab=rbind(outTab,

cbind(gene=gene,

HR=coxSummary$conf.int[,"exp(coef)"],

HR.95L=coxSummary$conf.int[,"lower .95"],

HR.95H=coxSummary$conf.int[,"upper .95"],

pvalue=coxP) )

}

}

write.table(outTab,file="uniCox.txt",sep="\t",row.names=F,quote=F)

surSigExp=rt[,sigGenes]

surSigExp=cbind(id=row.names(surSigExp),surSigExp)

write.table(surSigExp,file="uniSigExp.txt",sep="\t",row.names=F,quote=F)

bioForest=function(coxFile=null, forestFile=null){

rt <- read.table(coxFile, header=T, sep="\t", check.names=F, row.names=1)

gene <- rownames(rt)

hr <- sprintf("%.3f",rt$"HR")

hrLow <- sprintf("%.3f",rt$"HR.95L")

hrHigh <- sprintf("%.3f",rt$"HR.95H")

Hazard.ratio <- paste0(hr,"(",hrLow,"-",hrHigh,")")

pVal <- ifelse(rt$pvalue<0.001, "<0.001", sprintf("%.3f", rt$pvalue))

pdf(file=forestFile, width=6.6, height=4.5)

n <- nrow(rt)

nRow <- n+1

ylim <- c(1,nRow)

layout(matrix(c(1,2),nc=2),width=c(3,2.5))

xlim = c(0,3)

par(mar=c(4,2.5,2,1))

plot(1,xlim=xlim,ylim=ylim,type="n",axes=F,xlab="",ylab="")

text.cex=0.8

text(0,n:1,gene,adj=0,cex=text.cex)

text(1.5-0.5*0.2,n:1,pVal,adj=1,cex=text.cex);text(1.5-0.5*0.2,n+1,'pvalue',cex=text.cex,font=2,adj=1)

text(3.1,n:1,Hazard.ratio,adj=1,cex=text.cex);text(3.1,n+1,'Hazard ratio',cex=text.cex,font=2,adj=1)

par(mar=c(4,1,2,1),mgp=c(2,0.5,0))

xlim = c(0,max(as.numeric(hrLow),as.numeric(hrHigh)))

plot(1,xlim=xlim,ylim=ylim,type="n",axes=F,ylab="",xaxs="i",xlab="Hazard ratio")

arrows(as.numeric(hrLow),n:1,as.numeric(hrHigh),n:1,angle=90,code=3,length=0.05,col="darkblue",lwd=2.5)

abline(v=1,col="black",lty=2,lwd=2)

boxcolor = ifelse(as.numeric(hr) > 1, "red", "green")

points(as.numeric(hr), n:1, pch = 15, col = boxcolor, cex=1.5)

axis(1)

dev.off()

}

bioForest(coxFile="uniCox.txt", forestFile="forest.pdf")

**7. Prognostic LncRNA heatmap and boxplot**

#if (!requireNamespace("BiocManager", quietly = TRUE))

# install.packages("BiocManager")

#BiocManager::install("limma")

#install.packages("pheatmap")

#install.packages("reshape2")

#install.packages("ggpubr")

library(limma)

library(pheatmap)

library(reshape2)

library(ggpubr)

lncFile="uniSigExp.txt"

expFile="lncRNA.txt"

setwd("")

rt=read.table(expFile, header=T, sep="\t", check.names=F)

rt=as.matrix(rt)

rownames(rt)=rt[,1]

exp=rt[,2:ncol(rt)]

dimnames=list(rownames(exp),colnames(exp))

data=matrix(as.numeric(as.matrix(exp)),nrow=nrow(exp),dimnames=dimnames)

data=avereps(data)

data=data[rowMeans(data)>0,]

lncRNA=read.table(lncFile, header=T, sep="\t", check.names=F, row.names=1)

data=data[colnames(lncRNA)[3:ncol(lncRNA)],]

exp=data

group=sapply(strsplit(colnames(data),"\\-"), "[", 4)

group=sapply(strsplit(group,""), "[", 1)

group=gsub("2", "1", group)

conNum=length(group[group==1])

treatNum=length(group[group==0])

sampleType=c(rep(1,conNum), rep(2,treatNum))

sigVec=c()

for(i in row.names(data)){

test=wilcox.test(data[i,] ~ sampleType)

pvalue=test$p.value

Sig=ifelse(pvalue<0.001,"***",ifelse(pvalue<0.01,"**",ifelse(pvalue<0.05,"*","")))

sigVec=c(sigVec, paste0(i, Sig))

}

row.names(data)=sigVec

Type=c(rep("Normal",conNum), rep("Tumor",treatNum))

names(Type)=colnames(data)

Type=as.data.frame(Type)

data=log2(data+1)

pdf("heatmap.pdf", width=7.5, height=4.7)

pheatmap(data,

annotation=Type,

color = colorRampPalette(c(rep("blue",5), "white", rep("red",5)))(100),

cluster_cols =F,

cluster_rows =T,

scale="row",

show_colnames=F,

show_rownames=T,

fontsize=6,

fontsize_row=7,

fontsize_col=6)

dev.off()

exp=as.data.frame(t(exp))

exp=cbind(exp, Type=sampleType)

exp$Type=ifelse(exp$Type==1, "Normal", "Tumor")

data=melt(exp, id.vars=c("Type"))

colnames(data)=c("Type", "Gene", "Expression")

p=ggboxplot(data, x="Gene", y="Expression", color = "Type",

ylab="Gene expression",

xlab="",

legend.title="Type",

palette = c("blue", "red"),

width=1)

p=p+rotate_x_text(60)

p1=p+stat_compare_means(aes(group=Type),

method="wilcox.test",

symnum.args=list(cutpoints = c(0, 0.001, 0.01, 0.05, 1), symbols = c("***", "**", "*", " ")),

label = "p.signif")

pdf(file="boxplot.pdf", width=7.5, height=5)

print(p1)

dev.off()

**8. subtype cluster**

#if (!requireNamespace("BiocManager", quietly = TRUE))

# install.packages("BiocManager")

#BiocManager::install("ConsensusClusterPlus")

#if (!requireNamespace("BiocManager", quietly = TRUE))

# install.packages("BiocManager")

#BiocManager::install("limma")

library(limma)

library(ConsensusClusterPlus)

workDir="D:\\biowolf\\m6aLnc\\14.cluster"

setwd(workDir)

rt=read.table("uniSigExp.txt", header=T, sep="\t", check.names=F, row.names=1)

data=rt[,(3:ncol(rt))]

data=t(data)

maxK=9

results=ConsensusClusterPlus(data,

maxK=maxK,

reps=50,

pItem=0.8,

pFeature=1,

title=workDir,

clusterAlg="km",

distance="euclidean",

seed=123456,

plot="png")

clusterNum=2

cluster=results[[clusterNum]][["consensusClass"]]

write.table(cluster, file="cluster.txt", sep="\t", quote=F, col.names=F)

**9. survival analysis of subtype cluster**

#install.packages("survival")

#install.packages("survminer")

library(survival)

library(survminer)

clusterFile="cluster.txt"

cliFile="time.txt"

setwd("")

cluster=read.table(clusterFile, header=F, sep="\t", check.names=F, row.names=1)

cli=read.table(cliFile, header=T, sep="\t", check.names=F, row.names=1)

colnames(cli)=c("futime", "fustat")

cli$futime=cli$futime/365

sameSample=intersect(row.names(cluster), row.names(cli))

rt=cbind(cli[sameSample,], Cluster=cluster[sameSample,])

rt$Cluster=paste0("Cluster", rt$Cluster)

length=length(levels(factor(rt$Cluster)))

diff=survdiff(Surv(futime, fustat) ~ Cluster, data = rt)

pValue=1-pchisq(diff$chisq, df=length-1)

if(pValue<0.001){

pValue="p<0.001"

}else{

pValue=paste0("p=",sprintf("%.03f",pValue))

}

fit <- survfit(Surv(futime, fustat) ~ Cluster, data = rt)

#print(surv_median(fit))

bioCol=c("#0066FF","#FF0000","#FF9900","#6E568C","#7CC767","#223D6C","#D20A13","#FFD121","#088247","#11AA4D")

bioCol=bioCol[1:length]

surPlot=ggsurvplot(fit,

data=rt,

conf.int=F,

pval=pValue,

pval.size=6,

legend.title="Cluster",

legend.labs=levels(factor(rt[,"Cluster"])),

legend = c(0.8, 0.8),

font.legend=10,

xlab="Time(years)",

break.time.by = 1,

palette = bioCol,

surv.median.line="hv",

risk.table=T,

cumevents=F,

risk.table.height=.25)

pdf(file="survival.pdf",onefile = FALSE,width=7,height=5.5)

print(surPlot)

dev.off()

**10. Heatmap of subtype cluster and clinical feature**

#install.packages("pheatmap")

library(pheatmap)

ClusterFile="cluster.txt"

cliFile="clinical.txt"

expFile="uniSigExp.txt"

setwd("")

Cluster=read.table(ClusterFile, header=F, sep="\t", check.names=F, row.names=1)

colnames(Cluster)=c("Cluster")

Cluster=Cluster[order(Cluster$Cluster),,drop=F]

cli=read.table(cliFile, header=T, sep="\t", check.names=F, row.names=1)

samSample=intersect(row.names(Cluster), row.names(cli))

Cluster=Cluster[samSample,"Cluster",drop=F]

cli=cli[samSample,,drop=F]

Type=cbind(Cluster, cli)

Type$Cluster=paste0("Cluster", Type$Cluster)

sigVec=c("Cluster")

for(clinical in colnames(Type[,2:ncol(Type)])){

data=Type[c("Cluster", clinical)]

colnames(data)=c("Cluster", "clinical")

data=data[(data[,"clinical"]!="unknow"),]

tableStat=table(data)

stat=chisq.test(tableStat)

pvalue=stat$p.value

Sig=ifelse(pvalue<0.001,"***",ifelse(pvalue<0.01,"**",ifelse(pvalue<0.05,"*","")))

sigVec=c(sigVec, paste0(clinical, Sig))

#print(paste(clinical, pvalue, Sig, sep="\t"))

}

colnames(Type)=sigVec

exp=read.table(expFile, header=T, sep="\t", check.names=F, row.names=1)

data=t(exp[,(3:ncol(exp))])

data=data[,row.names(Type)]

colorList=list()

#Type=Type[apply(Type,1,function(x)any(is.na(match('unknow',x)))),,drop=F]

bioCol=c("#0066FF","#FF0000","#FF9900","#ed1299", "#0dbc21", "#246b93", "#cc8e12", "#d561dd", "#c93f00",

"#ce2523", "#f7aa5d", "#9ed84e", "#39ba30", "#6ad157", "#373bbf", "#a1ce4c", "#ef3bb6", "#d66551",

"#1a918f", "#7149af", "#ff66fc", "#2927c4", "#57e559" ,"#8e3af4" ,"#f9a270" ,"#22547f", "#db5e92",

"#4aef7b", "#e86502", "#99db27", "#e07233", "#8249aa","#cebb10", "#03827f", "#931635", "#ff523f",

"#edd05e", "#6f25e8", "#0dbc21", "#167275", "#280f7a", "#6373ed", "#5b910f" ,"#7b34c1" ,"#0cf29a" ,"#d80fc1",

"#dd27ce", "#07a301", "#ddd53e", "#391c82", "#2baeb5","#925bea", "#09f9f5", "#63ff4f")

j=0

for(cli in colnames(Type[,1:ncol(Type)])){

cliLength=length(levels(factor(Type[,cli])))

cliCol=bioCol[(j+1):(j+cliLength)]

j=j+cliLength

names(cliCol)=levels(factor(Type[,cli]))

if("unknow" %in% levels(factor(Type[,cli]))){

cliCol["unknow"]="grey75"}

colorList[[cli]]=cliCol

}

data=log2(data+1)

pdf("heatmap.pdf", width=7.5, height=5)

pheatmap(data,

annotation=Type,

annotation_colors = colorList,

color = colorRampPalette(c(rep("blue",5), "white", rep("red",5)))(100),

cluster_cols =F,

cluster_rows =T,

scale="row",

show_colnames=F,

show_rownames=T,

fontsize=6,

fontsize_row=6,

fontsize_col=6)

dev.off()

**11 immune cells infiltration**

#install.packages('e1071')

#if (!requireNamespace("BiocManager", quietly = TRUE))

# install.packages("BiocManager")

#BiocManager::install("preprocessCore")

#if (!requireNamespace("BiocManager", quietly = TRUE))

# install.packages("BiocManager")

#BiocManager::install("limma")

library("limma")

expFile="symbol.txt"

setwd("")

rt=read.table(expFile, header=T, sep="\t", check.names=F)

rt=as.matrix(rt)

rownames(rt)=rt[,1]

exp=rt[,2:ncol(rt)]

dimnames=list(rownames(exp),colnames(exp))

data=matrix(as.numeric(as.matrix(exp)),nrow=nrow(exp),dimnames=dimnames)

data=avereps(data)

data=data[rowMeans(data)>0,]

v <-voom(data, plot=F, save.plot=F)

out=v$E

out=rbind(ID=colnames(out), out)

write.table(out,file="uniq.symbol.txt",sep="\t",quote=F,col.names=F)

source("m6aLnc19.CIBERSORT.R")

results=CIBERSORT("ref.txt", "uniq.symbol.txt", perm=1000, QN=TRUE)

**12. difference analysis of immune cell and subtype cluster**

#if (!requireNamespace("BiocManager", quietly = TRUE))

# install.packages("BiocManager")

#BiocManager::install("limma")

#install.packages("ggpubr")

library(limma)

library(ggpubr)

immuneFile="CIBERSORT-Results.txt"

cluFile="cluster.txt"

pFilter=0.05

setwd("")

immune=read.table(immuneFile, header=T, sep="\t", check.names=F, row.names=1)

immune=immune[immune[,"P-value"]<pFilter,]

immune=as.matrix(immune[,1:(ncol(immune)-3)])

group=sapply(strsplit(row.names(immune),"\\-"), "[", 4)

group=sapply(strsplit(group,""), "[", 1)

group=gsub("2", "1", group)

immune=immune[group==0,]

row.names(immune)=gsub("(.*?)\\-(.*?)\\-(.*?)\\-(.*?)\\-.*", "\\1\\-\\2\\-\\3", row.names(immune))

immune=avereps(immune)

cluster=read.table(cluFile, header=F, sep="\t", row.names=1, check.names=F)

sameSample=intersect(row.names(immune), row.names(cluster))

immune1=immune[sameSample,,drop=F]

cluster1=cluster[sameSample,,drop=F]

colnames(cluster1)=c("Cluster")

data=cbind(immune1, cluster1)

data$Cluster=paste0("Cluster", data$Cluster)

type=levels(factor(data[,"Cluster"]))

data$Cluster=factor(data$Cluster, levels=type)

comp=combn(type, 2)

my_comparisons=list()

for(i in 1:ncol(comp)){my_comparisons[[i]]<-comp[,i]}

bioCol=c("#0066FF","#FF0000","#FF9900","#6E568C","#7CC767","#223D6C","#D20A13","#FFD121","#088247","#11AA4D")

bioCol=bioCol[1:length(levels(factor(data$Cluster)))]

for(i in colnames(data)[1:(ncol(data)-1)]){

boxplot=ggboxplot(data, x="Cluster", y=i, fill="Cluster",

xlab="",

ylab=i,

legend.title="Cluster",

palette=bioCol

)+

stat_compare_means(comparisons=my_comparisons)

pdf(file=paste0(i, ".pdf"), width=5, height=4.5)

print(boxplot)

dev.off()

}

**13. Tumor microenvironment**

#library(utils)

#rforge <- "http://r-forge.r-project.org"

#install.packages("estimate", repos=rforge, dependencies=TRUE)

#if (!requireNamespace("BiocManager", quietly = TRUE))

# install.packages("BiocManager")

#BiocManager::install("limma")

library(limma)

library(estimate)

inputFile="symbol.txt"

setwd("")

rt=read.table(inputFile, header=T, sep="\t", check.names=F)

rt=as.matrix(rt)

rownames(rt)=rt[,1]

exp=rt[,2:ncol(rt)]

dimnames=list(rownames(exp), colnames(exp))

data=matrix(as.numeric(as.matrix(exp)), nrow=nrow(exp), dimnames=dimnames)

data=avereps(data)

group=sapply(strsplit(colnames(data),"\\-"), "[", 4)

group=sapply(strsplit(group,""), "[", 1)

group=gsub("2", "1", group)

data=data[,group==0]

out=data[rowMeans(data)>0,]

out=rbind(ID=colnames(out), out)

write.table(out, file="uniq.symbol.txt", sep="\t", quote=F, col.names=F)

filterCommonGenes(input.f="uniq.symbol.txt",

output.f="commonGenes.gct",

id="GeneSymbol")

estimateScore(input.ds="commonGenes.gct",

output.ds="estimateScore.gct")

scores=read.table("estimateScore.gct", skip=2, header=T, check.names=F)

rownames(scores)=scores[,1]

scores=t(scores[,3:ncol(scores)])

rownames(scores)=gsub("\\.", "\\-", rownames(scores))

scores=scores[,1:3]

out=rbind(ID=colnames(scores), scores)

write.table(out,file="scores.txt",sep="\t",quote=F,col.names=F)

**14. Differential analysis of tumor microenvironment and** **subtype cluster**

#if (!requireNamespace("BiocManager", quietly = TRUE))

# install.packages("BiocManager")

#BiocManager::install("limma")

#install.packages("ggpubr")

library(limma)

library(ggpubr)

scoreFile="scores.txt"

cluFile="cluster.txt"

setwd("")

score=read.table(scoreFile, header=T, sep="\t", check.names=F, row.names=1)

row.names(score)=gsub("(.*?)\\-(.*?)\\-(.*?)\\-(.*?)\\-.*", "\\1\\-\\2\\-\\3", row.names(score))

score=avereps(score)

cluster=read.table(cluFile, header=F, sep="\t", row.names=1, check.names=F)

sameSample=intersect(row.names(score), row.names(cluster))

score1=score[sameSample,,drop=F]

cluster1=cluster[sameSample,,drop=F]

colnames(cluster1)=c("Cluster")

data=cbind(score1, cluster1)

data$Cluster=paste0("Cluster", data$Cluster)

type=levels(factor(data[,"Cluster"]))

data$Cluster=factor(data$Cluster, levels=type)

comp=combn(type, 2)

my_comparisons=list()

for(i in 1:ncol(comp)){my_comparisons[[i]]<-comp[,i]}

bioCol=c("#0066FF","#FF0000","#FF9900","#6E568C","#7CC767","#223D6C","#D20A13","#FFD121","#088247","#11AA4D")

bioCol=bioCol[1:length(levels(factor(data$Cluster)))]

for(i in colnames(data)[1:(ncol(data)-1)]){

boxplot=ggboxplot(data, x="Cluster", y=i, fill="Cluster",

xlab="",

ylab=i,

legend.title="Cluster",

palette=bioCol

)+

stat_compare_means(comparisons=my_comparisons)

pdf(file=paste0(i, ".pdf"), width=5, height=4.5)

print(boxplot)

dev.off()

}

**15. Prognostic model construction**

#install.packages("survival")

#install.packages("caret")

#install.packages("glmnet")

#install.packages("survminer")

#install.packages("timeROC")

library(survival)

library(caret)

library(glmnet)

library(survminer)

library(timeROC)

setwd("")

rt=read.table("uniSigExp.txt", header=T, sep="\t", check.names=F, row.names=1)

rt$futime[rt$futime<=0]=0.003

for(i in 1:1000){

inTrain<-createDataPartition(y=rt[,3], p=0.5, list=F)

train<-rt[inTrain,]

test<-rt[-inTrain,]

trainOut=cbind(id=row.names(train), train)

testOut=cbind(id=row.names(test), test)

x=as.matrix(train[,c(3:ncol(train))])

y=data.matrix(Surv(train$futime,train$fustat))

fit <- glmnet(x, y, family = "cox", maxit = 1000)

cvfit <- cv.glmnet(x, y, family="cox", maxit = 1000)

coef <- coef(fit, s = cvfit$lambda.min)

index <- which(coef != 0)

actCoef <- coef[index]

lassoGene=row.names(coef)[index]

geneCoef=cbind(Gene=lassoGene, Coef=actCoef)

if(nrow(geneCoef)<2){next}

trainFinalGeneExp=train[,lassoGene]

#myFun=function(x){crossprod(as.numeric(x), actCoef)}

#trainScore=apply(trainFinalGeneExp, 1, myFun)

trainScore=predict(cvfit, newx=as.matrix(train[,c(3:ncol(train))]), s="lambda.min", type="response")

outCol=c("futime", "fustat", lassoGene)

risk=as.vector(ifelse(trainScore>median(trainScore), "high", "low"))

train=cbind(train[,outCol], riskScore=as.vector(trainScore), risk)

trainRiskOut=cbind(id=rownames(train), train)

testFinalGeneExp=test[,lassoGene]

#testScore=apply(testFinalGeneExp, 1, myFun)

testScore=predict(cvfit, newx=as.matrix(test[,c(3:ncol(test))]), s="lambda.min", type="response")

outCol=c("futime", "fustat", lassoGene)

risk=as.vector(ifelse(testScore>median(trainScore), "high", "low"))

test=cbind(test[,outCol], riskScore=as.vector(testScore), risk)

testRiskOut=cbind(id=rownames(test), test)

diff=survdiff(Surv(futime, fustat) ~risk, data=train)

pValue=1-pchisq(diff$chisq, df=1)

diffTest=survdiff(Surv(futime, fustat) ~risk, data=test)

pValueTest=1-pchisq(diffTest$chisq, df=1)

predictTime=1

roc=timeROC(T=train$futime, delta=train$fustat,

marker=trainScore, cause=1,

weighting='aalen',

times=c(predictTime), ROC=TRUE)

rocTest=timeROC(T=test$futime, delta=test$fustat,

marker=testScore, cause=1,

weighting='aalen',

times=c(predictTime), ROC=TRUE)

if((pValue<0.01) & (roc$AUC[2]>0.7) & (pValueTest<0.05) & (rocTest$AUC[2]>0.65)){

write.table(trainOut,file="train.data.txt",sep="\t",quote=F,row.names=F)

write.table(testOut,file="test.data.txt",sep="\t",quote=F,row.names=F)

pdf("lambda.pdf")

plot(fit, xvar = "lambda", label = TRUE)

dev.off()

pdf("cvfit.pdf")

plot(cvfit)

abline(v=log(c(cvfit$lambda.min,cvfit$lambda.1se)),lty="dashed")

dev.off()

write.table(geneCoef, file="geneCoef.txt", sep="\t", quote=F, row.names=F)

write.table(trainRiskOut,file="trainRisk.txt",sep="\t",quote=F,row.names=F)

write.table(testRiskOut,file="testRisk.txt",sep="\t",quote=F,row.names=F)

allRiskOut=rbind(trainRiskOut, testRiskOut)

write.table(allRiskOut,file="allRisk.txt",sep="\t",quote=F,row.names=F)

break

}

}

**16. The survival curves**

#install.packages("survival")

#install.packages("survminer")

library(survival)

library(survminer)

setwd("D:\\biowolf\\m6aLnc\\27.survival")

bioSurvival=function(inputFile=null,outFile=null){

rt=read.table(inputFile, header=T, sep="\t")

diff=survdiff(Surv(futime, fustat) ~risk,data = rt)

pValue=1-pchisq(diff$chisq,df=1)

if(pValue<0.001){

pValue="p<0.001"

}else{

pValue=paste0("p=",sprintf("%.03f",pValue))

}

fit <- survfit(Surv(futime, fustat) ~ risk, data = rt)

surPlot=ggsurvplot(fit,

data=rt,

conf.int=T,

pval=pValue,

pval.size=6,

legend.title="Risk",

legend.labs=c("High risk", "Low risk"),

xlab="Time(years)",

break.time.by = 1,

palette=c("red", "blue"),

risk.table=TRUE,

risk.table.title="",

risk.table.col = "strata",

risk.table.height=.25)

pdf(file=outFile,onefile = FALSE,width = 6.5,height =5.5)

print(surPlot)

dev.off()

}

bioSurvival(inputFile="trainRisk.txt", outFile="trainSurv.pdf")

bioSurvival(inputFile="testRisk.txt", outFile="testSurv.pdf")

**17. Risk curve**

#install.packages("pheatmap")

library(pheatmap)

setwd("")

bioRiskPlot=function(inputFile=null,riskScoreFile=null,survStatFile=null,heatmapFile=null){

rt=read.table(inputFile, header=T, sep="\t", check.names=F, row.names=1)

rt=rt[order(rt$riskScore),] #????riskScore????Ʒ????

riskClass=rt[,"risk"]

lowLength=length(riskClass[riskClass=="low"])

highLength=length(riskClass[riskClass=="high"])

lowMax=max(rt$riskScore[riskClass=="low"])

line=rt[,"riskScore"]

line[line>10]=10

pdf(file=riskScoreFile, width=7, height=4)

plot(line, type="p", pch=20,

xlab="Patients (increasing risk socre)", ylab="Risk score",

col=c(rep("green",lowLength),rep("red",highLength)) )

abline(h=lowMax,v=lowLength,lty=2)

legend("topleft", c("High risk", "Low Risk"),bty="n",pch=19,col=c("red","green"),cex=1.2)

dev.off()

color=as.vector(rt$fustat)

color[color==1]="red"

color[color==0]="green"

pdf(file=survStatFile, width=7, height=4)

plot(rt$futime, pch=19,

xlab="Patients (increasing risk socre)", ylab="Survival time (years)",

col=color)

legend("topleft", c("Dead", "Alive"),bty="n",pch=19,col=c("red","green"),cex=1.2)

abline(v=lowLength,lty=2)

dev.off()

rt1=rt[c(3:(ncol(rt)-2))]

rt1=t(rt1)

annotation=data.frame(type=rt[,ncol(rt)])

rownames(annotation)=rownames(rt)

pdf(file=heatmapFile, width=7, height=4)

pheatmap(rt1,

annotation=annotation,

cluster_cols = FALSE,

cluster_rows = FALSE,

show_colnames = F,

scale="row",

color = colorRampPalette(c(rep("green",3.5), "white", rep("red",3.5)))(50),

fontsize_col=3,

fontsize=7,

fontsize_row=8)

dev.off()

}

bioRiskPlot(inputFile="trainRisk.txt",riskScoreFile="train.riskScore.pdf",survStatFile="train.survStat.pdf",heatmapFile="train.heatmap.pdf")

bioRiskPlot(inputFile="testRisk.txt",riskScoreFile="test.riskScore.pdf",survStatFile="test.survStat.pdf",heatmapFile="test.heatmap.pdf")

**18. The receiver operating characteristic**(ROC) **curve**

#install.packages("survival")

#install.packages("survminer")

#install.packages("timeROC")

library(survival)

library(survminer)

library(timeROC)

setwd("")

bioROC=function(inputFile=null, rocFile=null){

predictTime=1

rt=read.table(inputFile, header=T, sep="\t")

ROC_rt=timeROC(T=rt$futime, delta=rt$fustat,

marker=rt$riskScore, cause=1,

weighting='aalen',

times=c(predictTime), ROC=TRUE)

pdf(file=rocFile, width=5, height=5)

plot(ROC_rt, time=predictTime, col='red', title=FALSE, lwd=2)

legend('bottomright', cex=1.3,

paste0('AUC=',sprintf("%.03f",ROC_rt$AUC[2])),

col="white", lwd=1, bty = 'n')

dev.off()

}

bioROC(inputFile="trainRisk.txt",rocFile="train.ROC.pdf")

bioROC(inputFile="testRisk.txt",rocFile="test.ROC.pdf")

**19. Risk and clinical correlation heatmap**

#if (!requireNamespace("BiocManager", quietly = TRUE))

# install.packages("BiocManager")

#BiocManager::install("limma")

#install.packages("pheatmap")

library(limma)

library(pheatmap)

ClusterFile="cluster.txt"

cliFile="clinical.txt"

riskFile="allRisk.txt"

scoreFile="scores.txt"

setwd("")

Cluster=read.table(ClusterFile, header=F, sep="\t", check.names=F, row.names=1)

colnames(Cluster)=c("Cluster")

cli=read.table(cliFile, header=T, sep="\t", check.names=F, row.names=1)

risk=read.table(riskFile, header=T, sep="\t", check.names=F, row.names=1)

score=read.table(scoreFile, header=T, sep="\t", check.names=F, row.names=1)

row.names(score)=gsub("(.*?)\\-(.*?)\\-(.*?)\\-(.*?)\\-.*", "\\1\\-\\2\\-\\3", row.names(score))

score=avereps(score)

samSample=intersect(row.names(Cluster), row.names(cli))

Cluster=Cluster[samSample,"Cluster",drop=F]

cli=cli[samSample,,drop=F]

risk=risk[samSample,,drop=F]

score=score[samSample,,drop=F]

score[,"ImmuneScore"]=ifelse(score[,"ImmuneScore"]>median(score[,"ImmuneScore"]), "High", "Low")

data=cbind(risk, Cluster, score[,"ImmuneScore",drop=F], cli)

data=data[order(data$riskScore),,drop=F]

Type=data[,(ncol(risk):ncol(data))]

exp=data[,(3:(ncol(risk)-2))]

Type$Cluster=paste0("Cluster", Type$Cluster)

sigVec=c("risk")

for(clinical in colnames(Type[,2:ncol(Type)])){

data=Type[c("risk", clinical)]

colnames(data)=c("risk", "clinical")

data=data[(data[,"clinical"]!="unknow"),]

tableStat=table(data)

stat=chisq.test(tableStat)

pvalue=stat$p.value

Sig=ifelse(pvalue<0.001,"***",ifelse(pvalue<0.01,"**",ifelse(pvalue<0.05,"*","")))

sigVec=c(sigVec, paste0(clinical, Sig))

#print(paste(clinical, pvalue, Sig, sep="\t"))

}

colnames(Type)=sigVec

colorList=list()

#Type=Type[apply(Type,1,function(x)any(is.na(match('unknow',x)))),,drop=F]

bioCol=c("#FF0000","#0066FF","#0066FF","#FF0000","#FF9900","#ed1299", "#0dbc21", "#246b93", "#cc8e12", "#d561dd", "#c93f00",

"#ce2523", "#f7aa5d", "#9ed84e", "#39ba30", "#6ad157", "#373bbf", "#a1ce4c", "#ef3bb6", "#d66551",

"#1a918f", "#7149af", "#ff66fc", "#2927c4", "#57e559" ,"#8e3af4" ,"#f9a270" ,"#22547f", "#db5e92",

"#4aef7b", "#e86502", "#99db27", "#e07233", "#8249aa","#cebb10", "#03827f", "#931635", "#ff523f",

"#edd05e", "#6f25e8", "#0dbc21", "#167275", "#280f7a", "#6373ed", "#5b910f" ,"#7b34c1" ,"#0cf29a" ,"#d80fc1",

"#dd27ce", "#07a301", "#ddd53e", "#391c82", "#2baeb5","#925bea", "#09f9f5", "#63ff4f")

j=0

for(cli in colnames(Type[,1:ncol(Type)])){

cliLength=length(levels(factor(Type[,cli])))

cliCol=bioCol[(j+1):(j+cliLength)]

j=j+cliLength

names(cliCol)=levels(factor(Type[,cli]))

if("unknow" %in% levels(factor(Type[,cli]))){

cliCol["unknow"]="grey75"}

colorList[[cli]]=cliCol

}

pdf("heatmap.pdf", height=6, width=9)

pheatmap(t(exp),

annotation=Type,

annotation_colors = colorList,

color = colorRampPalette(c(rep("blue",5), "white", rep("red",5)))(100),

cluster_cols =F,

cluster_rows =F,

scale="row",

show_colnames=F,

show_rownames=T,

fontsize=6,

fontsize_row=7,

fontsize_col=6)

dev.off()

**20. Boxplot of risk and clinical correlation**

#if (!requireNamespace("BiocManager", quietly = TRUE))

# install.packages("BiocManager")

#BiocManager::install("limma")

#install.packages("ggpubr")

library(limma)

library(ggpubr)

ClusterFile="cluster.txt"

cliFile="clinical.txt"

riskFile="allRisk.txt"

scoreFile="scores.txt"

setwd("")

Cluster=read.table(ClusterFile, header=F, sep="\t", check.names=F, row.names=1)

colnames(Cluster)=c("Cluster")

cli=read.table(cliFile, header=T, sep="\t", check.names=F, row.names=1)

risk=read.table(riskFile, header=T, sep="\t", check.names=F, row.names=1)

score=read.table(scoreFile, header=T, sep="\t", check.names=F, row.names=1)

row.names(score)=gsub("(.*?)\\-(.*?)\\-(.*?)\\-(.*?)\\-.*", "\\1\\-\\2\\-\\3", row.names(score))

score=avereps(score)

samSample=intersect(row.names(Cluster), row.names(cli))

Cluster=Cluster[samSample,"Cluster",drop=F]

cli=cli[samSample,,drop=F]

risk=risk[samSample,,drop=F]

score=score[samSample,,drop=F]

score[,"ImmuneScore"]=ifelse(score[,"ImmuneScore"]>median(score[,"ImmuneScore"]), "High", "Low")

data=cbind(risk, Cluster, score[,"ImmuneScore",drop=F], cli)

rt=data[order(data$riskScore),,drop=F]

rt=rt[,((ncol(risk)-1):ncol(rt))]

rt=rt[,-2]

rt$Cluster=paste0("Cluster", rt$Cluster)

for(clinical in colnames(rt[,2:ncol(rt)])){

data=rt[c("riskScore", clinical)]

colnames(data)=c("riskScore", "clinical")

data=data[(data[,"clinical"]!="unknow"),]

group=levels(factor(data$clinical))

data$clinical=factor(data$clinical, levels=group)

comp=combn(group,2)

my_comparisons=list()

for(i in 1:ncol(comp)){my_comparisons[[i]]<-comp[,i]}

boxplot=ggboxplot(data, x="clinical", y="riskScore", color="clinical",

xlab=clinical,

ylab="Risk score",

legend.title=clinical,

add = "jitter")+

stat_compare_means(comparisons = my_comparisons)

pdf(file=paste0(clinical, ".pdf"), width=5.5, height=5)

print(boxplot)

dev.off()

}

**21. Single sample gene set enrichment analysis**

#if (!requireNamespace("BiocManager", quietly = TRUE))

# install.packages("BiocManager")

#BiocManager::install("limma")

#if (!requireNamespace("BiocManager", quietly = TRUE))

# install.packages("BiocManager")

#BiocManager::install("GSVA")

#if (!requireNamespace("BiocManager", quietly = TRUE))

# install.packages("BiocManager")

#BiocManager::install("GSEABase")

library(GSVA)

library(limma)

library(GSEABase)

setwd("C:\\biowolf\\pyroptosis\\32.ssGSEA")

immuneScore=function(expFile=null, gmtFile=null, project=null){

rt=read.table(expFile, header=T, sep="\t", check.names=F)

rt=as.matrix(rt)

rownames(rt)=rt[,1]

exp=rt[,2:ncol(rt)]

dimnames=list(rownames(exp),colnames(exp))

mat=matrix(as.numeric(as.matrix(exp)),nrow=nrow(exp),dimnames=dimnames)

mat=avereps(mat)

mat=mat[rowMeans(mat)>0,]

geneSet=getGmt(gmtFile, geneIdType=SymbolIdentifier())

ssgseaScore=gsva(mat, geneSet, method='ssgsea', kcdf='Gaussian', abs.ranking=TRUE)

normalize=function(x){

return((x-min(x))/(max(x)-min(x)))}

ssgseaOut=normalize(ssgseaScore)

ssgseaOut=rbind(id=colnames(ssgseaOut),ssgseaOut)

write.table(ssgseaOut, file=paste0(project, ".score.txt"), sep="\t", quote=F, col.names=F)

}

immuneScore(expFile="TCGA.normalize.txt", gmtFile="immune.gmt", project="TCGA")

immuneScore(expFile="GEO.normalize.txt", gmtFile="immune.gmt", project="GEO")

**22. Immune differential analysis**

#if (!requireNamespace("BiocManager", quietly = TRUE))

# install.packages("BiocManager")

#BiocManager::install("limma")

#install.packages("reshape2")

#install.packages("ggpubr")

library(limma)

library(reshape2)

library(ggpubr)

setwd("")

scoreCor=function(riskFile=null, scoreFile=null, project=null){

data=read.table(scoreFile, header=T, sep="\t", check.names=F, row.names=1)

data=t(data)

risk=read.table(riskFile, header=T, sep="\t", check.names=F, row.names=1)

sameSample=intersect(row.names(data),row.names(risk))

data=data[sameSample,,drop=F]

risk=risk[sameSample,,drop=F]

rt=cbind(data,risk[,c("riskScore","risk")])

rt=rt[,-(ncol(rt)-1)]

immCell=c("aDCs","B_cells","CD8+_T_cells","DCs","iDCs","Macrophages",

"Mast_cells","Neutrophils","NK_cells","pDCs","T_helper_cells",

"Tfh","Th1_cells","Th2_cells","TIL","Treg")

rt1=rt[,c(immCell,"risk")]

data=melt(rt1,id.vars=c("risk"))

colnames(data)=c("Risk","Type","Score")

data$Risk=factor(data$Risk, levels=c("low","high"))

p=ggboxplot(data, x="Type", y="Score", color = "Risk",

xlab="",ylab="Score",add = "none",palette = c("blue","red") )

p=p+rotate_x_text(50)

p=p+stat_compare_means(aes(group=Risk),symnum.args=list(cutpoints = c(0, 0.001, 0.01, 0.05, 1), symbols = c("***", "**", "*", "")),label = "p.signif")

pdf(file=paste0(project,".immCell.pdf"), width=7, height=6)

print(p)

dev.off()

immFunction=c("APC_co_inhibition","APC_co_stimulation","CCR",

"Check-point","Cytolytic_activity","HLA","Inflammation-promoting",

"MHC_class_I","Parainflammation","T_cell_co-inhibition",

"T_cell_co-stimulation","Type_I_IFN_Reponse","Type_II_IFN_Reponse")

rt1=rt[,c(immFunction,"risk")]

data=melt(rt1,id.vars=c("risk"))

colnames(data)=c("Risk","Type","Score")

data$Risk=factor(data$Risk, levels=c("low","high"))

p=ggboxplot(data, x="Type", y="Score", color = "Risk",

xlab="",ylab="Score",add = "none",palette = c("blue","red") )

p=p+rotate_x_text(50)

p=p+stat_compare_means(aes(group=Risk),symnum.args=list(cutpoints = c(0, 0.001, 0.01, 0.05, 1), symbols = c("***", "**", "*", "")),label = "p.signif")

pdf(file=paste0(project,".immFunction.pdf"), width=7, height=6)

print(p)

dev.off()

}

scoreCor(riskFile="trainRisk.txt", scoreFile="TCGA.score.txt", project="TCGA")

**23. Immune correlation analysis**

#if (!requireNamespace("BiocManager", quietly = TRUE))

# install.packages("BiocManager")

#BiocManager::install("limma")

#install.packages("ggplot2")

#install.packages("ggpubr")

#install.packages("ggExtra")

library(limma)

library(ggplot2)

library(ggpubr)

library(ggExtra)

immFile="CIBERSORT-Results.txt"

riskFile="allRisk.txt"

pFilter=0.05

setwd("")

immune=read.table(immFile, header=T, sep="\t", check.names=F, row.names=1)

immune=immune[immune[,"P-value"]<pFilter,]

immune=as.matrix(immune[,1:(ncol(immune)-3)])

group=sapply(strsplit(row.names(immune),"\\-"), "[", 4)

group=sapply(strsplit(group,""), "[", 1)

group=gsub("2", "1", group)

immune=immune[group==0,]

row.names(immune)=gsub("(.*?)\\-(.*?)\\-(.*?)\\-(.*?)\\-.*", "\\1\\-\\2\\-\\3", row.names(immune))

immune=avereps(immune)

risk=read.table(riskFile, header=T, sep="\t", check.names=F, row.names=1)

sameSample=intersect(row.names(immune),row.names(risk))

immune1=immune[sameSample,]

risk1=risk[sameSample,]

outTab=data.frame()

x=as.numeric(risk1[,"riskScore"])

for(j in colnames(immune1)){

y=as.numeric(immune1[,j])

if(sd(y)>0.001){

df1=as.data.frame(cbind(x,y))

corT=cor.test(x,y,method="spearman")

cor=corT$estimate

pValue=corT$p.value

p1=ggplot(df1, aes(x, y)) +

xlab("Risk score")+ ylab(j)+

geom_point()+ geom_smooth(method="lm",formula=y~x) + theme_bw()+

stat_cor(method = 'spearman', aes(x =x, y =y))

if(pValue<pFilter){

pdf(file=paste0(j,".pdf"), width=5, height=4.6)

print(p1)

dev.off()

outTab=rbind(outTab,cbind(Cell=j, pValue))

}

}

}

write.table(outTab,file="immuneCor.result.txt",sep="\t",row.names=F,quote=F)

**24. Immune checkpoint differential analysis**

#if (!requireNamespace("BiocManager", quietly = TRUE))

# install.packages("BiocManager")

#BiocManager::install("limma")

#install.packages("ggplot2")

#install.packages("ggpubr")

library(limma)

library(reshape2)

library(ggplot2)

library(ggpubr)

expFile="symbol.txt"

riskFile="risk.txt"

geneFile="gene.txt"

setwd("")

rt=read.table(expFile, header=T, sep="\t", check.names=F)

rt=as.matrix(rt)

rownames(rt)=rt[,1]

exp=rt[,2:ncol(rt)]

dimnames=list(rownames(exp),colnames(exp))

data=matrix(as.numeric(as.matrix(exp)),nrow=nrow(exp),dimnames=dimnames)

data=avereps(data)

gene=read.table(geneFile, header=F, sep="\t", check.names=F)

sameGene=intersect(row.names(data),as.vector(gene[,1]))

data=t(data[sameGene,])

data=log2(data+1)

group=sapply(strsplit(row.names(data),"\\-"),"[",4)

group=sapply(strsplit(group,""),"[",1)

group=gsub("2","1",group)

data=data[group==0,]

row.names(data)=gsub("(.*?)\\-(.*?)\\-(.*?)\\-(.*?)\\-.*","\\1\\-\\2\\-\\3",row.names(data))

data=avereps(data)

risk=read.table(riskFile, sep="\t", header=T, check.names=F, row.names=1)

sameSample=intersect(row.names(data),row.names(risk))

rt1=cbind(data[sameSample,],risk[sameSample,])

rt1=rt1[,c(sameGene,"risk")]

sigGene=c()

for(i in colnames(rt1)[1:(ncol(rt1)-1)]){

if(sd(rt1[,i])<0.001){next}

wilcoxTest=wilcox.test(rt1[,i] ~ rt1[,"risk"])

pvalue=wilcoxTest$p.value

if(wilcoxTest$p.value<0.05){

sigGene=c(sigGene, i)

}

}

sigGene=c(sigGene, "risk")

rt1=rt1[,sigGene]

rt1=melt(rt1,id.vars=c("risk"))

colnames(rt1)=c("risk","Gene","Expression")

group=levels(factor(rt1$risk))

rt1$risk=factor(rt1$risk, levels=c("low","high"))

comp=combn(group,2)

my_comparisons=list()

for(j in 1:ncol(comp)){my_comparisons[[j]]<-comp[,j]}

boxplot=ggboxplot(rt1, x="Gene", y="Expression", fill="risk",

xlab="",

ylab="Gene expression",

legend.title="Risk",

width=0.8,

palette = c("#0066FF", "#FF0000") )+

rotate_x_text(50)+

stat_compare_means(aes(group=risk),

method="wilcox.test",

symnum.args=list(cutpoints=c(0, 0.001, 0.01, 0.05, 1), symbols=c("***", "**", "*", "ns")), label="p.signif")

pdf(file="checkpoint.diff.pdf", width=8, height=5)

print(boxplot)

dev.off()

**25. Drug sensitivity analysis**

#if (!requireNamespace("BiocManager", quietly = TRUE))

# install.packages("BiocManager")

#BiocManager::install(c("car", "ridge", "preprocessCore", "genefilter", "sva"))

#install.packages("ggpubr")

library(limma)

library(ggpubr)

library(pRRophetic)

library(ggplot2)

set.seed(12345)

pFilter=0.001

expFile="symbol.txt"

riskFile="risk.all.txt"

setwd("")

allDrugs=c("A.443654", "A.770041", "ABT.263", "ABT.888", "AG.014699", "AICAR", "AKT.inhibitor.VIII", "AMG.706", "AP.24534", "AS601245", "ATRA", "AUY922", "Axitinib", "AZ628", "AZD.0530", "AZD.2281", "AZD6244", "AZD6482", "AZD7762", "AZD8055", "BAY.61.3606", "Bexarotene", "BI.2536", "BIBW2992", "Bicalutamide", "BI.D1870", "BIRB.0796", "Bleomycin", "BMS.509744", "BMS.536924", "BMS.708163", "BMS.754807", "Bortezomib", "Bosutinib", "Bryostatin.1", "BX.795", "Camptothecin", "CCT007093", "CCT018159", "CEP.701", "CGP.082996", "CGP.60474", "CHIR.99021", "CI.1040", "Cisplatin", "CMK", "Cyclopamine", "Cytarabine", "Dasatinib", "DMOG", "Docetaxel", "Doxorubicin", "EHT.1864", "Elesclomol", "Embelin", "Epothilone.B", "Erlotinib", "Etoposide", "FH535", "FTI.277", "GDC.0449", "GDC0941", "Gefitinib", "Gemcitabine", "GNF.2", "GSK269962A", "GSK.650394", "GW.441756", "GW843682X", "Imatinib", "IPA.3", "JNJ.26854165", "JNK.9L", "JNK.Inhibitor.VIII", "JW.7.52.1", "KIN001.135", "KU.55933", "Lapatinib", "Lenalidomide", "LFM.A13", "Metformin", "Methotrexate", "MG.132", "Midostaurin", "Mitomycin.C", "MK.2206", "MS.275", "Nilotinib", "NSC.87877", "NU.7441", "Nutlin.3a", "NVP.BEZ235", "NVP.TAE684", "Obatoclax.Mesylate", "OSI.906", "PAC.1", "Paclitaxel", "Parthenolide", "Pazopanib", "PD.0325901", "PD.0332991", "PD.173074", "PF.02341066", "PF.4708671", "PF.562271", "PHA.665752", "PLX4720", "Pyrimethamine", "QS11", "Rapamycin", "RDEA119", "RO.3306", "Roscovitine", "Salubrinal", "SB.216763", "SB590885", "Shikonin", "SL.0101.1", "Sorafenib", "S.Trityl.L.cysteine", "Sunitinib", "Temsirolimus", "Thapsigargin", "Tipifarnib", "TW.37", "Vinblastine", "Vinorelbine", "Vorinostat", "VX.680", "VX.702", "WH.4.023", "WO2009093972", "WZ.1.84", "X17.AAG", "X681640", "XMD8.85", "Z.LLNle.CHO", "ZM.447439")

rt = read.table(expFile, header=T, sep="\t", check.names=F)

rt=as.matrix(rt)

rownames(rt)=rt[,1]

exp=rt[,2:ncol(rt)]

dimnames=list(rownames(exp),colnames(exp))

data=matrix(as.numeric(as.matrix(exp)),nrow=nrow(exp),dimnames=dimnames)

data=avereps(data)

data=data[rowMeans(data)>0.5,]

group=sapply(strsplit(colnames(data),"\\-"), "[", 4)

group=sapply(strsplit(group,""), "[", 1)

group=gsub("2","1",group)

data=data[,group==0]

data=t(data)

rownames(data)=gsub("(.*?)\\-(.*?)\\-(.*?)\\-(.*)", "\\1\\-\\2\\-\\3", rownames(data))

data=avereps(data)

data=t(data)

riskRT=read.table(riskFile, header=T, sep="\t", check.names=F, row.names=1)

for(drug in allDrugs){

senstivity=pRRopheticPredict(data, drug, selection=1)

senstivity=senstivity[senstivity!="NaN"]

#senstivity[senstivity>quantile(senstivity,0.99)]=quantile(senstivity,0.99)

sameSample=intersect(row.names(riskRT), names(senstivity))

risk=riskRT[sameSample, "risk",drop=F]

senstivity=senstivity[sameSample]

rt=cbind(risk, senstivity)

rt$risk=factor(rt$risk, levels=c("low", "high"))

type=levels(factor(rt[,"risk"]))

comp=combn(type, 2)

my_comparisons=list()

for(i in 1:ncol(comp)){my_comparisons[[i]]<-comp[,i]}

test=wilcox.test(senstivity~risk, data=rt)

if(test$p.value<pFilter){

boxplot=ggboxplot(rt, x="risk", y="senstivity", fill="risk",

xlab="Risk",

ylab=paste0(drug, " senstivity (IC50)"),

legend.title="Risk",

palette=c("#0066FF","#FF0000")

)+

stat_compare_means(comparisons=my_comparisons)

pdf(file=paste0("durgSenstivity.", drug, ".pdf"), width=5, height=4.5)

print(boxplot)

dev.off()

}

}
